# Lymphocyte Gene Expression Signatures from Patients and Mouse Models of Hereditary Hemochromatosis Reveal a Function of HFE as a Negative Regulator of CD8^+^ T-Lymphocyte Activation and Differentiation *In Vivo*

**DOI:** 10.1371/journal.pone.0124246

**Published:** 2015-04-16

**Authors:** Mónica Costa, Eugénia Cruz, Susana Oliveira, Vladimir Benes, Tomi Ivacevic, Maria João Silva, Inês Vieira, Francisco Dias, Sónia Fonseca, Marta Gonçalves, Margarida Lima, Catarina Leitão, Martina U. Muckenthaler, Jorge Pinto, Graça Porto

**Affiliations:** 1 I_3_S - Instituto de Investigação e Inovação em Saúde, Universidade do Porto, Porto, Portugal; 2 Basic and Clinical Research on Iron Biology, IBMC—Instituto de Biologia Molecular e Celular, Universidade do Porto, Porto, Portugal; 3 Doctoral Program in Biomedicine, Faculdade de Medicina, Universidade do Porto, Porto, Portugal; 4 Clinical Hematology, Santo António Hospital—Centro Hospitalar do Porto, Porto, Portugal; 5 Genomics Core Facility, European Molecular Biology Laboratory, Heidelberg, Germany; 6 Advanced Flow Cytometry Unit, IBMC—Instituto de Biologia Molecular e Celular, Universidade do Porto, Porto, Portugal; 7 Departments of Pediatric Hematology, Oncology and Immunology, University of Heidelberg and Molecular Medicine Partnership Unit, Heidelberg, Germany; 8 Molecular Immunology and Pathology, ICBAS—Instituto de Ciências Biomédicas Abel Salazar, Universidade do Porto, Porto, Portugal; Lady Davis Institute for Medical Research/McGill University, CANADA

## Abstract

Abnormally low CD8^+^ T-lymphocyte numbers is characteristic of some patients with hereditary hemochromatosis (HH), a MHC-linked disorder of iron overload. Both environmental and genetic components are known to influence CD8^+^ T-lymphocyte homeostasis but the role of the HH associated protein HFE is still insufficiently understood. Genome-wide expression profiling was performed in peripheral blood CD8^+^ T lymphocytes from HH patients selected according to CD8^+^ T-lymphocyte numbers and from *Hfe* ^-/-^ mice maintained either under normal or high iron diet conditions. In addition, T-lymphocyte apoptosis and cell cycle progression were analyzed by flow cytometry in HH patients. HH patients with low CD8^+^ T-lymphocyte numbers show a differential expression of genes related to lymphocyte differentiation and maturation namely *CCR7*, *LEF1*, *ACTN1*, *NAA50*, *P2RY8* and *FOSL2*, whose expression correlates with the relative proportions of naïve, central and effector memory subsets. In addition, expression levels of *LEF1* and *P2RY8* in memory cells as well as the proportions of CD8^+^ T cells in G2/M cell cycle phase are significantly different in HH patients compared to controls. *Hfe* ^-/-^ mice do not show alterations in CD8^+^ T-lymphocyte numbers but differential gene response patterns. We found an increased expression of *S100a8* and *S100a9* that is most pronounced in high iron diet conditions. Similarly, CD8^+^ T lymphocytes from HH patients display higher S100a9 expression both at the mRNA and protein level. Altogether, our results support a role for HFE as a negative regulator of CD8^+^ T-lymphocyte activation. While the activation markers S100a8 and S100a9 are strongly increased in CD8^+^ T cells from both, *Hfe* ^-/-^ mice and HH patients, a differential profile of genes related to differentiation/maturation of CD8^+^ T memory cells is evident in HH patients only. This supports the notion that HFE contributes, at least in part, to the generation of low peripheral blood CD8^+^ T lymphocytes in HH.

## Introduction

Hereditary hemochromatosis is a common genetic disorder of iron overload where the vast majority of patients are homozygous for the C282Y mutation in *HFE*, a non-classical MHC-class I gene localized on chromosome 6 in strong linkage disequilibrium with the HLA-A locus [1,2]. The conformational change introduced by the C282Y mutation impairs the association of HFE with β(2)-microglobulin and consequently its expression at the cell surface [3]. As an integral part of a membrane-associated protein complex in hepatocytes, HFE is involved in the regulation of hepcidin, a hormone that controls systemic iron levels [4]. Whether HFE is also involved in T-lymphocyte signaling, is still unresolved.

Abnormalities in the pool of CD8^+^, but not CD4^+^, T lymphocytes have been consistently reported in HH patients [5]. Low numbers of CD8^+^ T lymphocytes in the peripheral blood [6–8] as well as in the liver [9] are associated with severe expression of iron overload. The low numbers of CD8^+^ T lymphocytes are mostly due to defects in the subpopulation of the effector memory T cells [10]. So far, functional studies have not been conclusive in terms of elucidating the nature of the CD8 defects in HH. Although decreased CD8-associated p56lck activity [11] and diminished cytotoxic activity [12] have been reported, other studies suggested a more activated profile of these cells namely a relative expansion of CD8^+^CD28^-^ T-cell populations, a high percentage of CD8^+^HLA-DR^+^ cells and an increased production of IL-4 and IL-10 [12,13]. A possible explanation for the activation profile of CD8^+^ T cells in HH may be found in a recent work where Reuben and co-workers propose that HFE has a role in antigen processing and presentation leading to an inhibition of CD8^+^ T-lymphocyte activation [14]. Their studies were based on several T-lymphocyte activation read-outs in cells transfected with wild type and mutated HFE molecules, but no evidence has been provided of an effect on antigen processing and presentation functions *in vivo*.

The anomalies of CD8^+^ T-lymphocyte numbers in HH could be a consequence of iron overload or a direct effect of HFE on the homeostatic regulation of this cell population. The finding that young, early diagnosed asymptomatic HH subjects already display a low CD8^+^ phenotype similar to their clinically affected family members, favors the idea of a predominantly primary genetic effect linked to the HFE mutation [7]. On the other hand, the fact that some HH patients despite the same HFE defect do not display the low CD8^+^ phenotype indicates that environmental and/or genetic factors must compensate for the anomalies in the CD8^+^ T-cell pool independently of HFE. It is known that modifications in gene expression concur to define the different properties of CD8^+^ T cells at different differentiation stages and therefore their homeostatic equilibrium [15]. We have shown that particular HLA-A alleles and haplotypes are significantly associated with CD8^+^ T-cell numbers in HH patients carrying the same *HFE* mutation [16,17] but they do not constitute a universal marker in all patients analyzed [18]. Studies are still pending trying to localize other relevant markers in the same chromosomal region. Genomic based studies, however, are not sufficient to distinguish the functional effect of HFE from that of other MHC-class I genes in strong linkage disequilibrium in the same chromosomal region.

In the present study we addressed the question whether HFE shapes the peripheral pools of CD8^+^ T lymphocytes. We applied two independent RNA-based genome wide approaches in isolated CD8^+^ T cells from HH patients homozygous for the C282Y *HFE* mutation and disease mouse models lacking the *HFE* gene (*Hfe* ^-/-^). While studies in HH patients revealed the impact of HFE on the expression of genes associated with the differentiation and maturation of peripheral CD8^+^ T-lymphocyte subsets, the transcriptional profile of isolated CD8^+^ T lymphocytes from *Hfe* ^-/-^ mice revealed alterations in CD8^+^ T-cell activation-related genes, a result also confirmed in peripheral blood lymphocytes from HH patients. Altogether, our results support a mechanistic role for *HFE* as a negative regulator of CD8^+^ T-lymphocyte activation *in vivo* and provide formal evidence, at least in part, to explain the characteristic low CD8 phenotype of HH patients.

## Results

### 1. A genome-wide transcriptional profile of CD8^+^ T lymphocytes from HH patients is indicative of the subpopulations’ differentiation/maturation states

A transcriptional gene profiling study of sorted CD8^+^ T lymphocytes from HH patients was performed to identify gene response patterns that may explain lower peripheral blood CD8^+^ T-lymphocyte numbers in patients with HH and severe iron overload. We took advantage of the known clinical and immune phenotypical variability in HH and selected 10 patients stratified in two distinct groups: group 1 (n = 6) shows a typical low CD8 phenotype (<300x10^3^/ml) (see Methods) hallmarked by a severe clinical expression of iron overload; group 2 (n = 4) shows normal/high CD8 phenotype (≥400x10^3^/ml) (see Methods) and very mild clinical expression of HH. Gene expression analysis identified a signature of 16 genes (7 up-regulated and 9 down-regulated) potentially associated with the CD8^+^ T-cell phenotype (Table 1). The magnitude of gene expression changes was small, generally less than 2-fold. Multiple variable correlation analysis among the 16 gene candidates identified two independent clusters of genes, which are functionally interlinked. One cluster contained the genes: chemokine C-C motif receptor 7 (*CCR7*), lymphoid enhancer-binding factor 1 (*LEF1*) and actinin alpha 1 (*ACTN1*), which are all down regulated in the group of patients with the low CD8 phenotype and are markers of CD8^+^ T-cell differentiation and/or maturation. The chemokine receptor CCR7 is a differentiation/maturation marker present in naïve (T_N_) and central memory (T_CM_) cells but absent in the effector memory (T_EM_) pool [19,20]. *LEF1* has been described as being down-regulated in naïve CD8^+^ T cells after antigen encounter and differentiation *in vivo* [21]. Of the three genes in this cluster, only *ACTN1* (HS1) was not previously described as a differentiation/maturation marker, but it is known to be involved in cytoskeletal remodeling and calcium mobilization, a fundamental process for T-cell activation [22]. A very distinct picture was observed for the second cluster of functionally related genes which included: N(alpha)-acetyltransferase 50 (*NAA50*), purinergic receptor P2Y (G-protein coupled, 8) (*P2RY8*) and FOS-like antigen 2, (*FOSL2*) that are all up-regulated in the group of patients with a low CD8 phenotype. This group of genes is involved in lymphocyte activation and expansion. Both *P2RY8* and *FOSL2* have been implicated as regulators of cell proliferation, differentiation, and malignant transformation [23,24] and *NAA50* is described as an anti-apoptotic molecule [25].

**Table 1:** Summary of differentially expressed genes in total CD8 T-lymphocytes in HH patients with a low CD8 phenotype (n = 6) relative to HH patients with a normal/ high CD8 phenotype (n = 4).

GeneBank accession #	Gene Symbol	Gene Description	Fold Change (Profile A vs Profile B)
NM_002123.3	*HLA-DQB1*	Major histocompatibility complex, class II, DQ beta1	Up 1.82
NM_002124.2	*HLA-DRB1*	Major histocompatibility complex, class II, DR beta1	Up 1.82
NM_005253	*FOSL2* ^a)^	FOS-like antigen 2	Up 1.59
BC096168	*HIST1H1E*	Histone cluster 1, H1e	Up 1.57
NM_178129	*P2RY8* ^a)^	Purinergic receptor P2Y, G-protein coupled, 8	Up 1.57
BC009288	*NR4A2* ^a)^	Nuclear receptor subfamily 4, group A, member 2	Up 1.52
BC012731	*NAA50* ^a)^	N(alpha)-acetyltransferase 50, NatE catalytic subunit	Up 1.51
NR_003330	*SNORD116-15*	Small nucleolar RNA, C/D box 116–15	Down 1.90
BC038982	*IGJ*	Immunoglobulin J polypeptide, linker protein for immunoglobulin alpha and mu polypeptides	Down 1.81
NR_002907	*SNORA73A*	Small nucleolar RNA, H/ACA box 73A	Down 1.74
BC035343	*CCR7* ^a)^	Chemokine (C-C motif) receptor 7	Down 1.68
DQ496098	*ACTN1* ^a)^	Actinin, alpha 1	Down 1.65
NR_003332	*SNORD116-17*	Small nucleolar RNA, C/D box 116–17	Down 1.57
NR_001290	*SNORD116-19*	Small nucleolar RNA, C/D box 116–19	Down 1.57
AF288571	*LEF1* ^a)^	Lymphoid enhancer-binding factor 1	Down 1.56
NM_019111.4	*HLA-DRA*	Major histocompatibility complex, class II, DR alpha	Down 1.52

For definition of low CD8 phenotype and normal/high CD8 phenotype see Methods.

a) Confirmed by custom designed real-time PCR primers

Besides the above described genes, a significant up regulation of the MHC class II genes *HLA*-*DQB1* and *HLA*-*DRB1*, as well as the histone cluster 1 gene *HIST1H1e*, was also observed in patients with a low CD8 phenotype, supporting the notion of an activation and cell cycle progression pattern of these cells [26].

In order to explore the significance of the results obtained in this screen, we analyzed the correlations of the expression levels of each gene with the clinical and immune phenotypic variables. These included the number of peripheral blood CD8^+^ T lymphocytes, including the differential pattern of CD8 T_N_, T_CM_ and T_EM_ cells as well as the iron overload profile, measured by the estimated total body iron stores (TBIS). The results are given in S1 Table. They strongly suggest that the differences observed in the gene expression of CD8^+^ T cells between the two HH groups reflect not only upon differences in the relative proportions of their CD8^+^ T-cell subsets but also upon differences in the activation profile of the cells. More specifically, the differentiation markers *CCR7*, *LEF1* and *ACTN1* correlated positively with the number of CD8^+^ T_N_ and T_CM_ cells in each patient, reflecting a direct impact of the relative proportion of these subpopulations in the total CD8^+^ pool expression pattern. In contrast, the activation markers *NAA50*, *P2RY8* and *FOSL2* were negatively correlated with the total number of CD8^+^ T_EM_ cells in each patient, indicating that subjects with a low CD8 phenotype show more activated effector memory cells. Curiously, variations in the mRNA expression levels of these activation genes had more impact on the total number of CD8^+^ T lymphocytes than the expression of the differentiation markers *CCR7*, *LEF1* and *ACTN1* (see data on S1 Table).

In general, the TBIS was most significantly correlated with the differentiation markers *CCR7*, *LEF1* and *ACTN1* (see also data on S1 Table) therefore not excluding an effect of iron overload on the most immature CD8^+^ T lymphocytes or vice-versa. It should be noted, however, that due to the strict selection criteria applied in this screen, it was not possible to discriminate the confounding effects of the CD8^+^ T-cell numbers and of the iron overload phenotypes, because the two variables are highly correlated (*R* ^*2*^ = 57%; *r* = -0.75, *p* = 0.0187).

In order to rule out the hypothesis of a bystander effect of systemic inflammation on the patients’ individual immunophenotypes, we retrieved historical measures of their C-reactive protein (CRP) serum levels and compared them with the CD8^+^ T-lymphocyte counts determined on the same day. No significant correlation was found between the two parameters (*R*-squared adjusted for d.f. = -1.19185 percent; *p* = 0.379), thus excluding any relevant effect of systemic inflammation on the patients’ immunophenotype.

### 2. Analysis of CD8^+^ T-lymphocyte subsets reveals the impact of HFE on the expression profile of central memory and effector memory cells

**Fig 1:**
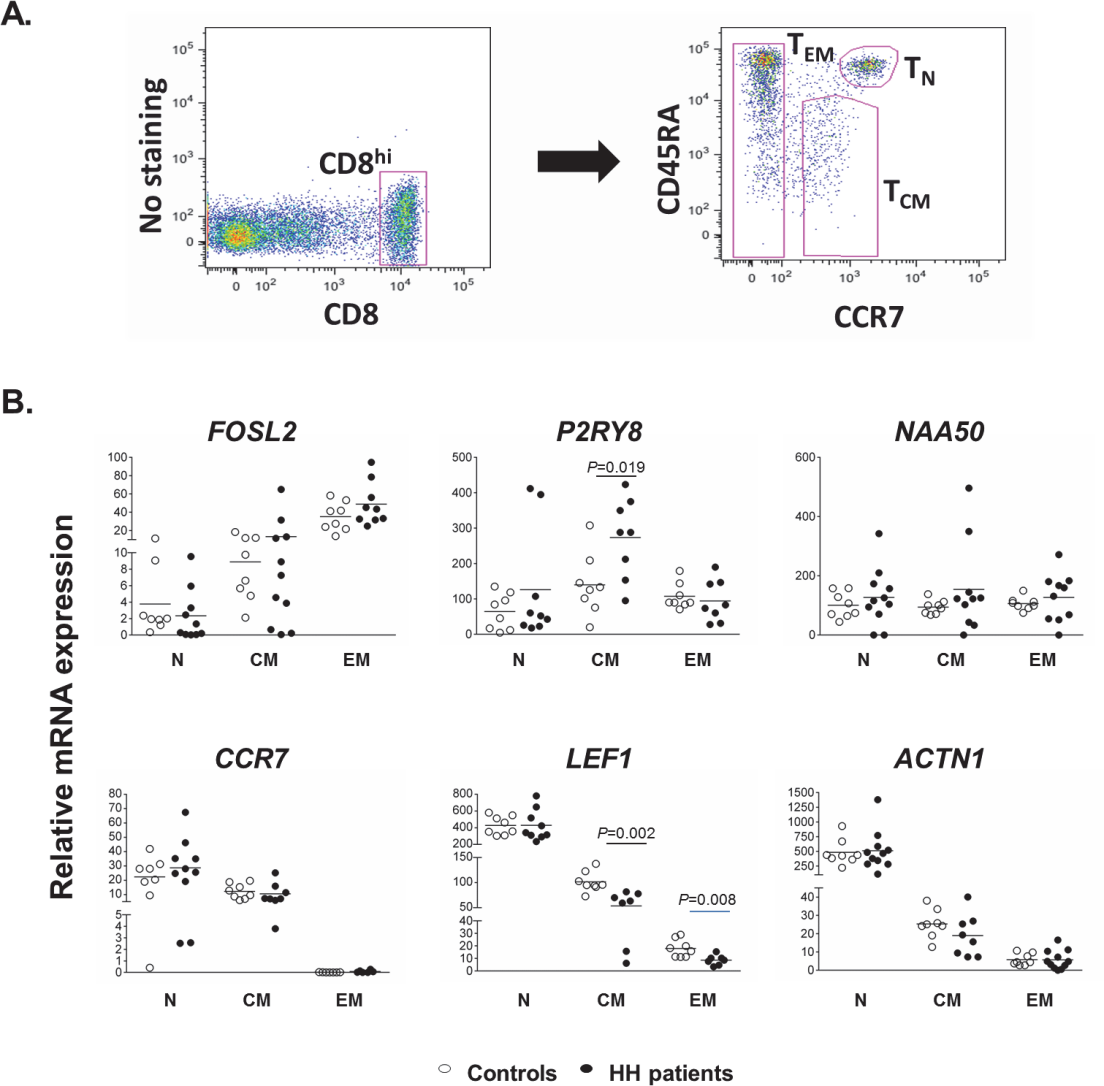
Gene expression analysis in CD8^+^ T-cell subpopulations. A) Gating strategy used to discriminate the CD8^+^ T subpopulations of naïve, central memory and effector memory cells. B) Relative mRNA expression levels of *CCR7*, *LEF1*, *ACTN1*, *FOSL2*, *P2RY8* and N*AA50* in isolated CD8^+^ T subpopulations. Statistical significant differences were calculated by T-test between HH patients and controls in each subpopulation.

As shown above, the transcriptional profiles of HH patients with low or normal/high CD8^+^ T-cell numbers not only highlight the differences in the relative proportions of their subsets but also reflect differences in the activation state of the cells. To understand if the gene expression profiles are informative beyond reflecting upon T-cell numbers, we next focused on the analysis of 6 genes (*CCR7*, *LEF1*, *ACTN1*, *FOSL2*, *P2RY8* and *NAA50*) in the different CD8^+^ T_N_, T_CM_ and T_EM_ compartments. Cells were sorted according to the gating strategy illustrated in Fig 1A, in blood samples from a group of 10 additional and unselected HH patients and 8 healthy control individuals. With this approach we aimed: i) to validate the results of the genome wide screen with independent HH samples ii) to test the impact of HFE on gene expression by comparing HH patients with normal healthy subjects and iii) to address if there was any additional impact of serum iron levels on the expression profiles in individual T-cell populations of HH patients, taking advantage of the fact that patients were at different stages of treatment and therefore showing a wide range of transferrin saturation values.

As illustrated in Fig 1B, the mRNA expression levels of *CCR7*, *LEF1* and *ACTN1* decreased from CD8^+^ T_N_ to T_EM_ cells, with the lowest values consistently observed in the T_EM_ subset. Once *CCR7* is a well-known maturation marker of T lymphocytes [19] it served as a non-anticipated positive control in our experiment. An opposite pattern was detected for *FOSL2*, which was up-regulated in the most differentiated lymphocyte subpopulation scrutinized, i.e. in T_EM_. *P2RY8*, in turn, showed the highest expression levels in T_CM_ cells, while *NAA50* was uniformly expressed in all CD8^+^ T-cell subsets. Of note, significant differences were observed in the profile of T_CM_ cells between HH patients and controls, *LEF1* being significantly decreased (*p* = 0.002) and *P2RY8* significantly increased (*p* = 0.019) in HH patients. In addition, *LEF1* in HH patients was also significantly decreased in T_EM_ (*p* = 0.008). These differences between HH patients and controls in the gene transcription of central and effector memory cells, suggest that the HFE defect may affect the homeostatic equilibrium of these particular populations without affecting the naïve T cells. Importantly, no significant correlations were found between the expression of any of the analyzed genes and the levels of circulating iron as measured by the transferrin saturation (data not shown) further supporting a primary effect of HFE on CD8^+^ T-lymphocyte signaling independently from circulating iron levels.

### 3. Apoptosis and cell cycle studies in CD4^+^ and CD8^+^ T lymphocytes from HH patients and controls

The fact that the genes found up regulated in patients with the lowest CD8^+^ counts were genes involved in activation and proliferation suggests that, in spite of constituting a small pool, these cells must be constantly activated to proliferate. The next question to address was therefore to investigate whether an altered apoptosis/proliferation balance of peripheral blood CD8^+^ T lymphocytes in HH patients can account for these observations. Since this is the first study analysing apoptosis and cell cycle profiles in T lymphocytes from HH patients, the study was extended to both CD4^+^ and CD8^+^ subpopulations of T lymphocytes.

#### 3.1 Apoptosis in CD8^+^ T lymphocytes correlates with systemic iron levels

In general, a highly significant difference was found between the CD4^+^ and CD8^+^ T-lymphocyte subpopulations in terms of the percentages of apoptotic cells (T Test *p* = 9.4x10^-8^, KS-Test *p* = 0.000002), with the average apoptosis percentage being 21.8% ±9.9% in CD4^+^ T cells, and 47.3% ±21.8% in CD8^+^ T cells. Apoptosis in CD4^+^ and CD8^+^ T cells was not influenced by gender but it was influenced by age in CD8^+^ T cells only (*R* ^*2*^ = 13.5%, *r* = 0.37, *p* = 0.0380). In general, apoptosis in each of these subsets (CD4^+^ or CD8^+^ T cells) was not statistically different between controls and HH patients and, in this later group, it was not influenced by the treatment status i.e. (intensive vs. maintenance treatment) (data not shown). In terms of association with total numbers, no significant correlations were found between apoptosis and either CD4^+^ or CD8^+^ T-cell total counts (cells/mm^3^) in both HH patients and controls, indicating that defective numbers in HH are not explained by increased apoptosis (data not shown). Nevertheless, the percentage of apoptosis in both CD8^+^ and CD4^+^ T cells in HH patients was significantly correlated with the systemic iron load parameters namely serum iron (respectively *R* ^*2*^ = 34.6%, *p* = 0.008 and *R* ^*2*^ = 20.5%, *p* = 0.046) and transferrin saturation (respectively *R* ^*2*^ = 32.9%, *p* = 0.006, and *R* ^*2*^ = 20.4%, *p* = 0.045).

#### 3.2 Peripheral blood CD8^+^ T lymphocytes from HH patients show an increased proportion of cells in the G2/M phase

Marked differences were observed between the populations of CD4^+^ and CD8^+^ T cells regarding the proportion of cells along the three phases of cell cycle, particularly in G2M and S (see data on Table 2). Regarding the differences between HH patients and controls, some significant differences could be observed independently of iron status (see also Table 2). In general, there were more lymphocyte in S and G2/M and less in G0/G1 phase in HH patients than in controls, both in CD4^+^ and CD8^+^. These differences reached statistical significance in G2/M with values in patients being 2.5 and 11 times higher respectively in CD8^+^ and CD4^+^ cells, and in G0/G1 for CD4^+^ T cells only, although this difference is less than 0.2% of their absolutes levels, probably reflecting the space occupied by the increased numbers of cells in G2/M. No significant correlation was found between the percentages of cells in each cell cycle phase and the percentage of cells in apoptosis, showing that the increase of cells in G2/M observed in patients is not a direct consequence of apoptosis but rather an independent step, possibly related with increased cell activation.

**Table 2:** Comparisons of CD4^+^ and CD8^+^ T-lymphocyte percentages in different cell cycle phases between HH patients and controls.

Cell Cycle Phase	Cell population	% in Controls	% in HH Patients	KS test^1^
**G0G1**	**CD4** ^**+**^ **T cells**	99.65 ± 0.16	99.41 ± 0.33	P = 0.0256
	**CD8** ^**+**^ **T cells**	98.86 ± 1.25	98.13 ± 1.69	n.s
	***KS test*** ^***2***^	0.0337	0.0047	
**S**	**CD4** ^**+**^ **T cells**	0.34 ± 0.16	0.49 ± 0.23	n.s
	**CD8** ^**+**^ **T cells**	0.94 ± 1.00	1.39 ±1.09	n.s
	***KS test*** ^***2***^	0.0337	0.0015	
**G2M**	**CD4** ^**+**^ **T cells**	0.01 ± 0.03	0.11 ± 0.14	P = 0.0217
	**CD8** ^**+**^ **T cells**	0.20 ± 0.36	0.49 ± 0.74	P = 0.0025
	***KS test*** ^***2***^	<0.0001	0.0135	

^1^ Kolmogorov-Smirnov Test comparing HH patients and controls

^2^ Kolmogorov-Smirnov Test comparing CD4^+^ T cells and CD8^+^ T cells

In summary, results of cell cycle positioning of total peripheral blood CD8^+^ T lymphocytes from HH patients support the notion that those cells constitute a highly dynamic population in homeostatic equilibrium, compatible with the activation profile revealed before with the gene expression data.

### 4. mRNA expression analysis in *Hfe* ^-/-^ and wild type mice supports the impact of HFE on the CD8^+^ T-lymphocyte activation profile

All previous results in HH patients suggesting an impact of HFE on the expression profile of CD8^+^ T cells were still confounded not only by the known phenotypic heterogeneity among patients, possibly influenced by other MHC linked genetic determinants [16,18] but also by the strong correlation between the CD8 phenotypes and the severity of iron overload [8]. In order to address the relative impact of HFE and iron overload on the CD8^+^ T-lymphocyte gene expression profile in the absence of such confounding variables, we used the HFE deficient mouse model (*Hfe* ^-/-^). We performed a differential genome-wide expression analysis comparing *Hfe* ^-/-^ and wild-type (wt) mice, in conditions of either normal or iron rich diet. Results showed that few genes were differently expressed in the CD8^+^ T cells of *Hfe* ^*-/-*^ mice, 66 in total: 37 up-regulated and 29 down-regulated under the standard normal iron condition. A few more genes were differently expressed under high iron diet, 78 in total: 67 up-regulated and 11 down-regulated. Lists of the transcripts differentially regulated in *Hfe* ^-/-^ in comparison with wild type C57BL/6 mice are provided in S2 and S3 Tables. Functional clustering analysis was performed to define categories among the differentially regulated genes. The results are presented as supplementary material in S4 Table.

**Fig 2:**
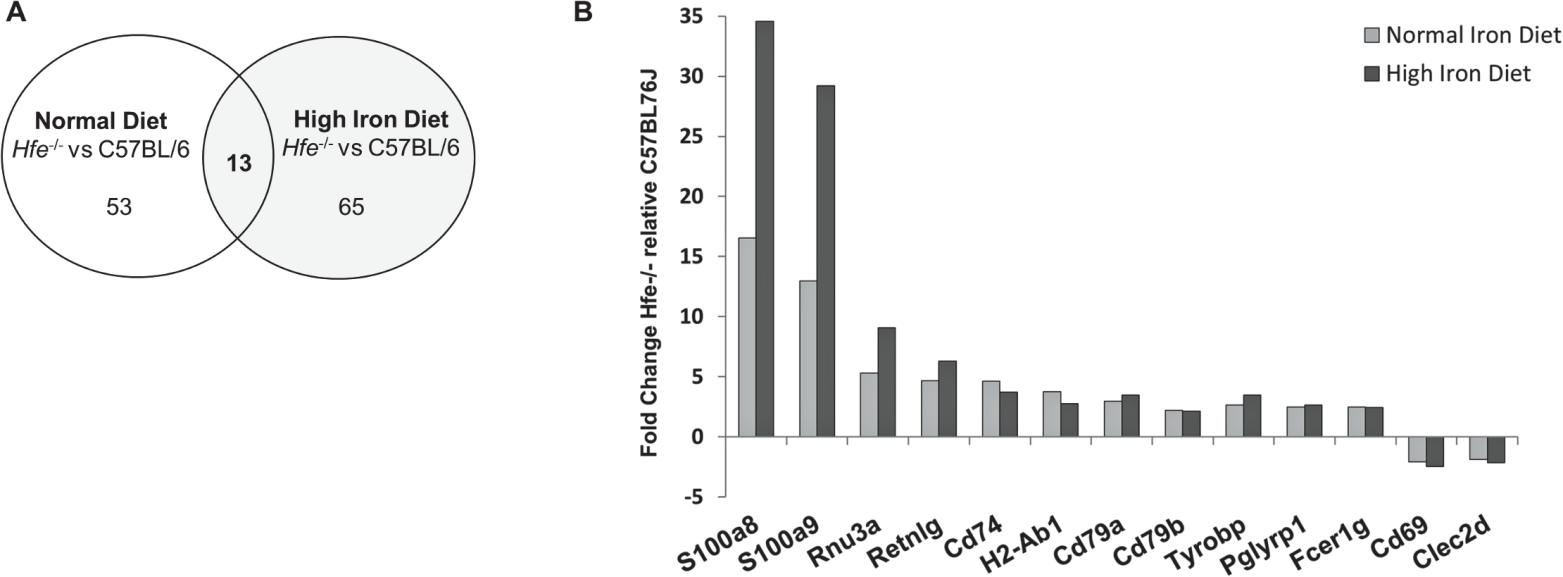
Genome-wide expression analysis of CD8^+^ T lymphocytes from *Hfe* ^-/-^ and wild type mice. A) Venn diagram with comparative analysis between the selected genes found to be differently regulated by the two genotypes under the same iron diet condition. B) Differential gene expression of *Hfe* ^-/-^ mice with normal and high iron diet relatively to C57BL/6 mice under the same iron diet conditions. Fold Change of the genes found to be differently expressed (T-test, *p*<0.05) and with fold change >1.8 are represented.

From all differentially expressed genes, only thirteen were found in common in both normal and high iron diet conditions (Fig 2A), suggesting an impact of HFE on the CD8 expression of these genes regardless of iron levels. Nevertheless, an additional effect of iron cannot be excluded. As illustrated in Fig 2B, the most striking differences were found for *S100a8* and *S100a9*, two calcium-binding proteins (calgranulins) involved in the regulation of several cellular processes such as cell cycle progression and differentiation [27,28]. These genes were increased in *Hfe* ^-/-^ mice under normal iron diet (fold-changes of 16.52 for *S100a8* and 12.96 for *S100a9*) but notably, the fold-change differences were even higher under high iron diet conditions (fold-changes of 34.58 for *S100a8* and 29.21 for *S100a9*).

#### 4.1 Correlational analysis of the differential expressed genes between Hfe^-/-^ and wild type mice

In order to understand the putative interaction of the calgranulins S100a8 and S100a9 with the other differentially expressed genes, we further analyzed expression data from all individual mice maintained in high iron diet conditions (the condition where differential gene expression was more marked) and performed multi-variable correlation analysis to identify the most significantly co-expressed genes. The results are given as supplementary material illustrated in S1 Fig. In general, correlations with *S100a9* were stronger than those observed with *S100a8*. The gene whose expression was most significantly correlated with *S100a9* was *Tyrobp* (*r* = 0.9931; *p* = 0.0001) which codes for the tyrosine kinase binding protein *DAP12* (DNAX activation protein 12). Interestingly, *DAP12* has been shown by others to be a marker of self-reactive, non-MHC restricted activated memory-phenotype CD8^+^ T cells, in contrast to the conventional CD8^+^ T cells [29]. Two other genes, *Retn1g* and *Fcer1g* were found strongly correlated with *DAP12* expression (*r* = 0.9756; *p* = 0.0009 and *r* = 0.9701; *p* = 0.0013, respectively) and, accordingly, they were the next most significantly correlated with *S100a9* expression (*r* = 0.9693; *p* = 0.0014 and *r* = 0.9558; *p* = 0.0029, respectively). *Retn1g* codes for a novel resistin-like molecule expressed in hematopoietic tissues and expected to have a cytokine-like role [30] and *Fcer1g* codes for the gama Fc membrane receptor (FcRγ) which, like *DAP12*, is a tyrosine kinase binding protein with a fundamental role in immune effector functions [31]. Two genes, *Cd69* and *Clec2d*, were the only inversely correlated with *S100a9* (*r* = -0.9764; *p*<0.0001 and *r* = -0.9421; *p* = 0.0005, respectively). These are respectively the members C and D of the C-type lectin domain family 2, a family of co-stimulatory molecules recently recognized as markers of very early T-cell activation [32]. In particular *Cd69* has been described as a marker of non-circulating resident (resting) memory CD8^+^ T cells, and characteristically absent in the recirculating central and effector memory CD8^+^ T-cell populations [33]. The only gene from the initial list of 13 that was not significantly correlated with *S100a9* at the individual level was *CD79a*. In summary, results of a significantly increased expression of *S100a9* in CD8^+^ T lymphocytes from *Hfe* ^-/-^ mice positively correlated with *DAP12* and negatively correlated with *Cd69* suggests that the effect of HFE on CD8 activation may target preferentially the central/effector memory cells, which are also the cells predominantly decreased in HH patients [10].

None of the genes found altered in HH patients with a low CD8 phenotype (*LEF1* and *P2RY8)* were significantly associated with the up regulation of calgranulins *S100a8* and *S100a9*, showing that the two models of differential gene expression are not equivalent. Hence the putative mechanisms involving HFE as a player in CD8^+^ T-cell activation in mice or those involving HFE as a player in the homeostatic equilibrium of CD8^+^ T-cell subsets in HH patients may be substantially different.

#### 4.2 Genes encoding proteins of iron metabolism are not altered in CD8^+^ T cells from Hfe^-/-^ mice

**Fig 3:**
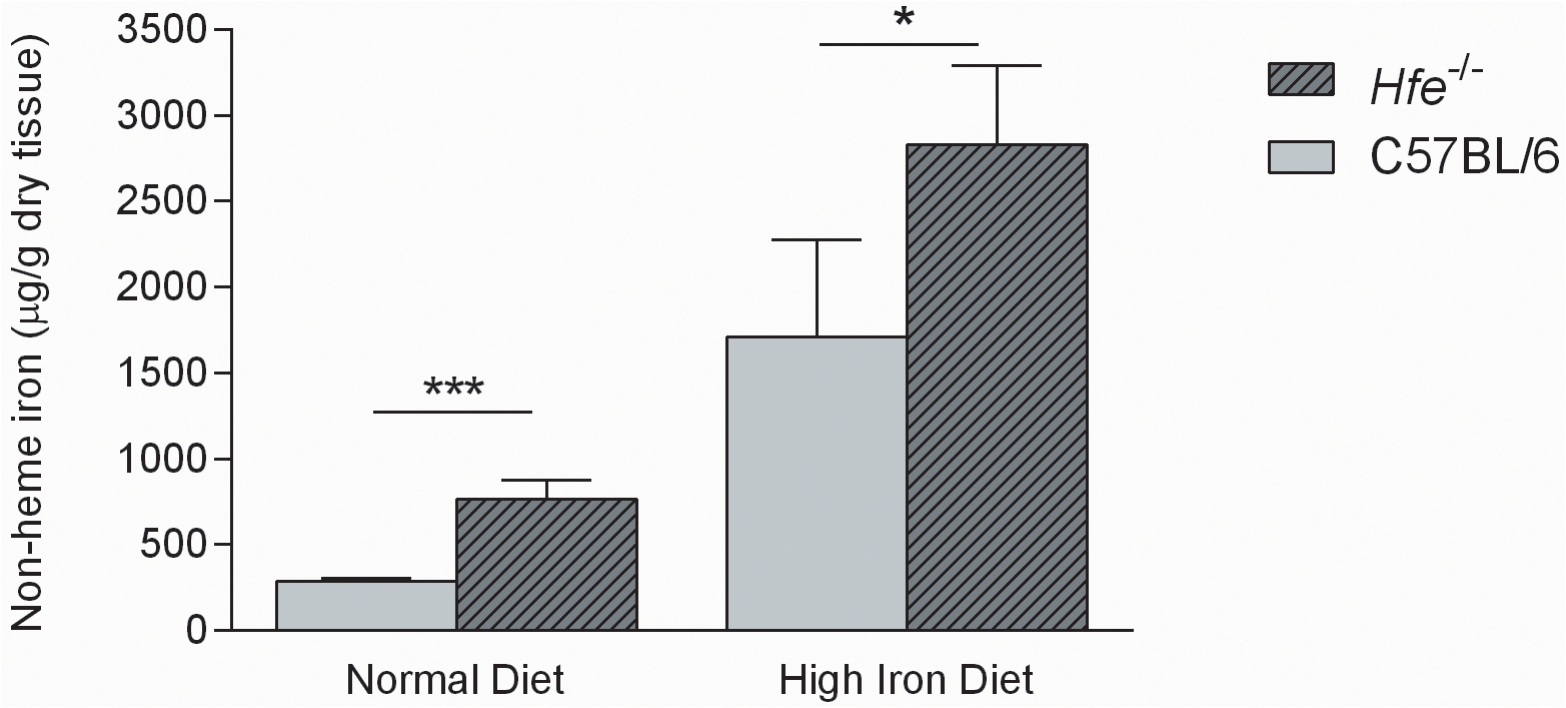
Hepatic iron concentration of *Hfe* ^-/-^ and C57BL/6 mice under a normal or a high-iron diet. Values are expressed as mean ± standard deviation. Statistical significant differences were calculated between mice genotype groups under the same diet condition (T-test * *P*<0.05 and ***P<0.001).

To assess whether iron impacts on the differential gene expression of *Hfe* ^-/-^ mice we analyzed the expression of genes related with iron metabolism in normal iron diet conditions, where the differences between the two mouse models in terms of iron loading were more marked (Fig 3). The expression of most iron related genes was, in general, very low in CD8^+^ T lymphocytes and not significantly different between the two genotypes except for lipocalin 2 (*Lcn2*) that was significantly up-regulated in *Hfe* ^-/-^ in comparison with wild-type (*p* = 0.0016). *Lcn*2 is an antimicrobial protein that acts by capturing and depleting bacterial siderophores and is known to have chemoattractant properties [34]. These results are shown as supplementary material in S5 Table. No significant correlations were found between the expression of any of the 13 genes differentially expressed in Hfe^-/-^ mice and the expression of the iron related genes (data not shown).

### 5. S100a9 mRNA and protein expression are increased in human peripheral blood CD8^+^ T lymphocytes from HH patients

**Fig 4:**
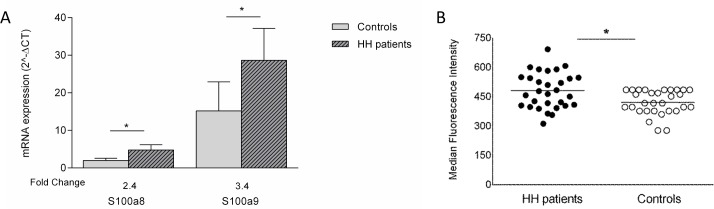
S100a8 and S100a9 expression in CD8^+^ T cells from HH patients and controls. A) *S100a8* and *S100a9* mRNA expression in isolated CD8^+^ T lymphocytes from HH patients and normal controls. Statistical significance was obtained with paired analysis of patient-control of the day using the non-parametric Wilcoxon test, *p<0.05. B) Median fluorescence intensity of S100a9 expression in CD8^+^ T cells from peripheral blood from HH patients and normal controls. Statistical significance was obtained with paired analysis of patient versus control of the day using the parametric T-test, *p<0.0001.

The previous findings that calgranulins are over-expressed in CD8^+^ T lymphocytes of *Hfe* ^-/-^ mice, prompted us to investigate these proteins in human patients with HH in comparison to normal controls. This was done first by accessing *S100a8* and *S100a9* expression at mRNA levels with qRT-PCR without any previously activation step. As shown in Fig 4A, *S100a8* and *S100a9* mRNA expression in isolated CD8^+^ T lymphocytes were significantly higher in HH patients than in controls with fold change of 2.4 for *S100a8* and 3.4 for *S100a9* (Wilcoxon paired test, *p* = 0.016 and *p* = 0.027 respectively). Although in the *Hfe* ^-/-^ mouse model these two proteins were similarly expressed, in humans *S100a9* gene expression was found to be 4.5x higher than *S100a8*. Due to this high expression, we next analyzed S100a9 protein level by flow cytometry.

The expression of S100a9 protein was evaluated by MFI (median fluorescence intensity) in total lymphocytes, CD8^+^ and CD4^+^ T subpopulations. In addition, the expression of S100a9 was also evaluated in neutrophils and monocytes that are known to express this protein and were used as positive controls (see Material and Methods). Paired T-Test comparisons of S100a9 MFI values in the several populations were performed between HH patients (n = 30) and controls (n = 13) tested in the same day under the same conditions. Neutrophils and monocytes had significantly (*p* = 0.013 and *p* = 0.005 respectively) higher S100a9 MFI values in HH patients (mean±SEM: 13090±1039 and 2947±181 respectively) in comparison with controls (mean±SEM: 10291±293 and 2435±80 respectively). The expression of S100a9 in total lymphocytes was also consistently higher in HH patients than in controls (mean±SEM: 481±15 in HH and 418±10 in controls, *p*<0.0001), although at a much lower level. Regarding the T-lymphocyte subsets, the expression of S100a9 in CD8^+^ T lymphocytes was significantly higher in HH patients in comparison to controls (mean±SEM: 481±17 in HH and 421±11 in controls, *p*<0.0001) (Fig 4B). The same result was obtained for CD4^+^ T cells (mean±SEM: 469±17 in HH and 388±9 in controls, *p*<0.0001). S100a9 expression in each cell type was not significantly correlated with the total numbers of the respective cells.

Altogether, results in both animal and human models of HH support a direct effect of HFE on the transcriptional profile of CD8^+^ T lymphocytes, HFE deficiency inducing the up regulation of the calgranulin S100a9. As an HFE-dependent effect, it is not surprising that calgranulins have not been found differentially expressed in the first genome-wide screen in patients, where all subjects carry the same *HFE* mutation. Hence, results of calgranulin expression do not constitute an explanation for the heterogeneity found in HH patients regarding the numbers and subsets of CD8^+^ T lymphocytes. These were better explained by changes in the signaling pathways described above with the results of the differential expression profiles in CD8 subsets.

## Discussion

In this paper we questioned whether or not HFE, a non-classical MHC-class I molecule, affects the triggering/signaling pathways of CD8^+^ T lymphocytes and if it contributes to the self-renewal and homeostasis of the three main CD8+ subpopulations of naïve, central memory and effector memory cells at the periphery. To do that, we used human and mouse models of HH, both lacking the cell surface expression of HFE. In a first step we analyzed the transcriptional profile of selected CD8^+^ T lymphocytes from HH patients homozygous for the C282Y *HFE* mutation. Because these patients share a common *HFE* defect but differ in the numbers of CD8^+^ T lymphocytes and subpopulations, this approach was expected to identify novel candidates associated with the CD8 phenotype independently of HFE. The signaling molecules *CCR7* and *LEF1*, which are normally down regulated in naïve cells after activation [21], as well as *ACTN1*, a calcium dependent remodeling molecule, were significantly decreased in patients with a low CD8 phenotype. Conversely, the expression of *FOSL2*, *P2RY8* and *NAA50* molecules known to be involved in processes of activation/expansions of lymphocytes, were significantly increased in patients with low CD8 phenotype. By mRNA analysis in sorted populations we confirmed the expression patterns of these genes in naïve, central memory and effector memory cells. While *CCR7* and *LEF1* were, as expected, mostly expressed in naïve cells we describe for the first time that and *ACTN1* is also mostly expressed in naïve cells, that *P2RY8* expression is preferentially observed in central memory and that *FOSL2* is preferentially expressed in effector memory cells. This general pattern of subset specific gene expression was found both in HH patients and controls (see Fig 1). Nevertheless, the expression of *LEF1* and *P2RY8* in central and effector memory CD8 subpopulations of HH patients differed significantly from controls, independently of their total cell numbers or serum iron levels. We interpret these results as evidence of an impact of HFE on the differentiation /maturation of CD8^+^ T lymphocytes, possibly in the conversion step of central memory into effector memory T cells described by Peixoto and co-workers with *in vitro* experiments [15]. The question remains, however, if a similar expression profile would be also observed in normal subjects displaying a low CD8 phenotype, in spite of a normal HFE. Amongst the healthy controls studied here (n = 8), only one subject displayed a low CD8 phenotype (total CD8+ T lymphocytes = 197x10^3^/ml; total T_EM_ T lymphocytes = 0.058 x10^3^/ml). But in contrast to HH patients, he had no evidence of a decreased *LEF1* or increased *P2RY8* expression in T_CM_ or T_EM_ subpopulations as compared to the other controls (data not shown), therefore not supporting a defect similar to that observed in HH. A formal proof to this concept, however, would imply an extended analysis of a large normal population, which was out of the scope of the present paper.

The observation that HH patients show an increased proportion of cells in G2/M phase in the periphery is compatible with the hypothesis of an increased activation state. In addition, the present study of apoptosis and cell cycle in human peripheral blood lymphocytes also brought into light a new aspect of lymphocyte biology which is the clear demonstration of marked differences in apoptosis and cell cycle progression between the subpopulations of CD4^+^ and CD8^+^ T lymphocytes. Although a higher percentage of apoptotic cells among the CD8^+^ relative to CD4^+^ T lymphocytes had been already observed in previous studies in the context of sepsis and pulmonary disease, its significance in normal physiological conditions had never been discussed [35,36]. This is a relevant finding deserving fully consideration in future studies of lymphocyte activation and proliferation in the clinical setting.

One pending question that could not be answered by the simple analysis of CD8 expression data in HH patients was the clarification of the complex interaction between HFE, CD8^+^ T-lymphocyte numbers and the severity of iron overload. As mentioned in the results section, a limitation of the genome-wide screen experiment in HH patients was the fact that, due to the strict selection criteria used, i.e., patients grouped according to exclusive patterns of CD8 phenotypes (low or normal/high) which are significantly associated with the iron overload profiles, it was not possible to distinguish the relative interactions of iron overload or CD8 numbers with the expression profiles. Nevertheless, for the subsequent analysis of expression of the different genes in sorted CD8^+^ T-cell subsets we used a non-selected group of patients who were at different stages of treatment showing a wide range of transferrin saturation values not related to the CD8 phenotype. Although there was, in general, a higher variation in the gene expression of the different CD8^+^ T-cell subsets in HH patients than in controls (see Fig 1B), we found no correlation with either transferrin saturation or total CD8 numbers, supporting the notion of a primary effect of HFE on CD8^+^ T-cell signaling, independent of actual circulating iron levels.

The above described impact of HFE on the CD8^+^ lymphocyte profile independently of iron levels does not exclude the hypothesis that CD8^+^ T lymphocytes may be equipped with some “sensing” system for iron. In this regard, it was interesting to observe a relationship between higher transferrin saturation and increased CD8^+^ T-cell apoptosis. Iron induced apoptosis had been already reported in human hepatocytes and rat neurons but it had never been described in human lymphocytes [37,38]. It could be speculated that in this way iron overload could contribute to the decreased numbers of CD8^+^ T cells in HH. However, a direct relationship between apoptosis and cell numbers was not found. The most plausible explanation is a compensatory increased activation of CD8^+^ T cells contributing to a “more dynamic” pool of lymphocytes in HH as shown by an increase in the number of cells in G2/M. We should bear in mind, however, that an increased number of cells in G2/M is not exclusively due to an increased number of mitotic cells but may also reflect an increased number of cells arrested in G2 for DNA repair, eventually in response to iron-induced oxidative injury. Of note, in a previous study we have shown that HH patients´ lymphocytes had lower DEB-induced chromosome instability possibly due to an adaptive response of the HH lymphocytes with increased DNA repair [39].

Once the question of the relationship of iron overload and the expression profile of CD8^+^ T lymphocytes could not be clarified in the human model of HH, we decided to address this question in animal models where genetic and environmental variables are controlled. For that purpose, we analyzed the transcriptional profile of CD8^+^ T lymphocytes from mice *Hfe* ^-/-^ in comparison to normal mice of the same genetic background, experiments performed in either normal or high iron diet conditions. Because all mice share the same MHC background, any putative modifier effect of other MHC related molecules could not be a variable here. The results revealed a set of differentially expressed genes between the two strains and three features deserve to be mentioned. First, the differentially expressed genes identified were mostly clustered in functional types related to lymphocyte signaling and activation, supporting the suggested inhibitory effect of wild-type HFE on CD8 activation [14]. Secondly, most of these differentially expressed genes differed in conditions of normal or high iron diet. Nevertheless, the observed impact of a high iron diet is only a partial effect because *Hfe* ^-/-^ mice are already constitutively iron overloaded. Finally the genes previously found altered in HH patients with a low CD8 phenotype were not altered in the *Hfe* ^-/-^ mouse model indicating that the mouse model does not completely recapitulate the phenotype of HH in humans, which is not surprising taking into consideration the fact that abnormalities in CD8^+^ T-cell numbers have never been described in these mice.

The most striking differences in *Hfe* ^-/-^ mice were observed for the expression *S100a8* and *S100a9*, belonging to the S100 family of proteins containing two canonical EF-hand calcium-binding motifs involved in the calcium dependent control of cell differentiation, cell cycle progression and growth [27,28]. The fold-change values of differential expression of these calgranulins between *Hfe* ^-/-^ and wild type mice was superior in mice fed in a high iron diet than for mice under a normal diet. Interestingly, a previously published genome wide mRNA expression study aimed to assess the effect of iron loading in muscle cells from mice also revealed that, among others, *S100a8* and *S100a9* were over expressed in iron overload conditions [40]. Further studies are needed to clarify the putative modulatory role of systemic iron on CD8^+^ T-cell activation.

Results of calgranulins expression in mice was next translated to the human clinical model of HH where patients with a non-functional HFE showed an increased expression in both the mRNA expression and intracytoplasmatic protein expression of S100a9 in CD8^+^ T lymphocytes. It should be reminded however that an HFE-dependent altered gene expression in CD8^+^ T lymphocytes does not necessarily mean that HFE is exerting its effect on the surface of these cells. On the contrary, it is more plausible to assume that an HFE-dependent alteration in antigen-presenting cells may indirectly affect the CD8^+^ T-cell signaling. In this context, it was interesting to observe that, at the protein level, an increased expression of S100a9 was also observed in other blood cell types which are known to express this protein in higher amounts, namely neutrophils and monocytes. The additional finding of a strong correlation between the expression of *S100a9* and the adaptor protein *DAP12* is also of particular interest and may open new avenues to better explore the pathways involved in the effect of HFE on CD8^+^ T-cell activation.

The putative role of iron on CD8^+^ T-lymphocyte activation and differentiation deserves some additional considerations. For many years we have reported results of a negative correlation between the numbers of CD8^+^ T lymphocytes and the severity of iron overload in HH [6,8,41], supporting the postulate that they may act as systemic “buffers” to protect against systemic iron toxicity [42–46]. Following that hypothesis, we have recently demonstrated the capacity of peripheral blood lymphocytes to uptake and process NTBI [47] and in that way to protect against tissue iron accumulation [48]. The mechanisms involved in NTBI transport and signaling in lymphocytes are still elusive. It is well known that non-classical MHC I molecules display different features from the classical ones, namely in their capacity to bind non peptide ligands [49]. In the recent paper by Reuben and co-workers on the inhibitory effect of HFE on CD8^+^ T-lymphocyte activation they excluded the interaction with TfR1 as a necessary step for HFE-mediated inhibition of MHC I presentation. It would be interesting to explore if HFE could affect NTBI uptake and in that way somehow influence lymphocyte activation.

The assumption of a mechanistic model of interactions between HFE, lymphocyte activation and S100a9 expression may have important implications for a better understanding of HH and its clinical consequences. We propose that the “low CD8 phenotype” in HH is the result of a homeostatic equilibrium of cells constantly triggered to activate and differentiate into more mature effector cells. This hypothesis is compatible with the concept first advanced by Reuben and co-workers that HFE is a negative regulator of CD8^+^ T-lymphocyte activation and that the lack of HFE may render CD8^+^T lymphocytes less tolerant to constant stimuli and more susceptible to autoimmune phenomena [14]. Notably, calgranulins have been described as damage-associated molecular pattern molecules (DAMPs) highly up regulated in various autoimmune disorders [28]. Recently Loser and colleagues provided clear evidence, in both animal and human autoimmune disorder models that local calgranulin production is essential for the induction of autoreactive CD8^+^ T cells and the development of systemic autoimmunity, an effect mediated via Toll-like receptor 4 (TLR4) signaling [28]. The idea that a normal surface HFE expression may help preventing the development of autoimmunity in homeostatic conditions [14] would imply that HH patients should be also more susceptible to autoimmune disorders. Although these are not commonly described in HH, one may recall here the fact that one of the most perturbing clinical features of HH is a severe arthropathy of still unknown pathogenesis that is not prevented by iron depletion, suggesting that other HFE-related mechanisms should be involved. Considering the recently described effect of S100a9 as an inflammation orchestrator in rheumatoid arthritis [28] and lupus erythematosus [50], it is tempting to speculate that an increased expression of S100a9 could also contribute to the arthropathy process in HH. More studies are certainly needed to address this question and to understand if there is any impact of activated CD8^+^ T lymphocytes on the pathogenesis of HH arthropathy, what is the role of calgranulins in that process and if they could constitute a promising novel therapeutic target.

## Methods

### Ethics statement

Animal care and procedures were in accordance with institutional guidelines. Conducted experiments in mice were approved by the IBMC.INEB Animal Ethics Committee, in accordance with the Portuguese Veterinary Director General guidelines. Regarding human studies, they were approved by the Institutional Ethical Committee of Santo António Hospital—Centro Hospitalar do Porto and written informed consent was obtained from all participants in accordance with the Declaration of Helsinki.

### Mice

Mice models used in this study were C57BL/6 supplied by Charles River and mice homozygous for the disruption of the *Hfe* gene (*Hfe* ^-/-^) generated under the same genetic background as described elsewhere [51]. They were females maintained at the IBMC’s Animal Care Facility fed *ad libitum* with the standard local rodent diet (Teklan 2014 Harlan with 175ppm of iron) until 14–15 weeks of age. After this, four C57BL/6 wild-type and four *Hfe* ^-/-^ mice were fed with the same diet until they were 16–17 weeks old consisting in the “normal iron diet” group. Other four C57BL/6 wild type and four *Hfe* ^-/-^ mice were fed, during one week, with a mix of 50% Harlan 2014 and 50% Harlan Iron Rich (supplemented with 2.5% carbonyl iron corresponding to 25000 ppm of iron) (TD.06700) following 100% Harlan Iron Rich one more week until they were 16–17 weeks old, consisting in the “high-iron diet” group. To confirm the validity of the different diet conditions, non-heme iron concentration was determined in the liver of each mouse (n = 16). As expected, significantly higher iron content was observed in the *Hfe* ^-/-^ mice in comparison with C57BL/6 under the same diet (Fig 3).

### Human subjects

All patients included in this study were diagnosed and are regularly followed-up at the Hemochromatosis Outpatient Clinic of Santo Antonio Hospital (Porto, Portugal) by the same dedicated clinician. Patients are all unrelated, and genetically characterized as homozygous for the C282Y mutation of the *HFE* gene. They were recruited to participate in the study in a consecutive mode at the time of their regular consultations. Retrospective clinical and laboratory data from patients were obtained from the clinical files under the responsibility of the clinician in charge. These included a) the individual iron overload profile at diagnosis estimated by the total body iron stores measured by quantitative phlebotomies [52], b) the actual iron parameters at the time of experiment measured by serum iron and transferrin saturation; c) the individual immune-phenotype with determinations of total CD8^+^ T-lymphocyte numbers as well as measures of the CD8^+^ T subsets of naïve, central memory and effector memory cells, and d) measures of CRP as a marker of systemic inflammation. For the purpose of phenotypic grouping, HH patients were classified into one of these two types: “the low CD8 phenotype” defined as patients with absolute CD8^+^ T-lymphocyte numbers consistently lower than 300x10^3^/ml in at least 5 serial determinations, and the “normal/high CD8 phenotype” defined as patients with CD8^+^ T-lymphocyte numbers consistently higher or equal than 400x10^3^/ml. These limits were defined based on the normal distribution of values described in the Portuguese control population considering the limit for the low CD8 phenotype as the 25% lower percentile and the limit for a normal/high CD8 phenotype the average value in controls [8,18]. Clinical and laboratory data from the patients have been published previously [2,8,10,41,46].

Healthy controls for the study were consecutively recruited amongst volunteer blood donors at the Blood Bank of Santo Antonio Hospital during their regular visits for blood donation.

### Experimental procedures

#### a) Genome-wide expression analysis of CD8^+^ T lymphocytes from HH patients

Twenty-four HH patients were selected for the present experiment which comprised two parts: a genome-wide gene expression screening, followed by a gene profiling of selected CD8^+^ T-cell subpopulations. In the first part (genome-wide gene expression screening) 10 patients were selected and stratified into the two subgroups of “low” (n = 6) or “normal/high” (n = 4) as defined above. As expected from previously described data [2,8,10,41,46], patients in the low group had characteristically a severe iron overload while in the other group subjects were asymptomatic. In the second part, 14 previously unselected patients were consecutively enrolled for gene expression profiling of sorted CD8^+^ T-cell subpopulations, independently of their CD8 phenotype or iron status. They were at different stages of treatment which allowed us to obtain a sample with a wide range of transferrin saturation values. A group of 8 healthy blood donors were used as controls.

For the purpose of genome-wide screening, CD8^+^ T lymphocytes from HH patients were positively selected from peripheral blood mononuclear cells (PBMCs) that were isolated from whole blood or buffy coat samples by density separation over Lymphoprep 1.077g/ml density gradient (Axis-Shield). CD8^+^ T-lymphocytes were positively selected from PBMCs (~3x10^7^ cells) by Magnetic-Activated Cell Sorting (MACS; Miltenyi Biotec), following the manufacturer’s instructions. Total RNA of MACS-purified CD8^+^ T cells was extracted using Mini RNeasy Plus *Kit* Mini (Qiagen, Valencia, CA) as recommended by the manufacturer. RNA concentration and purity were determined using optical density (OD) measurements at 260 and 280 nm. All the samples had an OD260/OD280 ratio of 1.95 or higher. In each experiment time total RNA concentration was normalized between patients and controls and converted into cDNA with NZY First-Strand cDNA Synthesis kit (Nzytech) as recommended by the manufacturer. Transcriptional profile of CD8^+^ T lymphocytes was assessed using the GeneChip Human Gene 1.0 ST array (Affymetrix). The RNA processing and hybridization steps, carried out at the Genomics Core facility of the European Molecular Biology Laboratory (EMBL, Heidelberg), were performed as recommended by the manufacturer. Upon filtering and normalization of the raw data, samples were grouped according to the CD8 low or normal/high profiles and subjected to a between-group analysis using the GeneSpring GX software (Agilent). Genes which exhibited at least a 1.5-fold change in expression were considered as up- or down-regulated. Quantitate real-time PCR was used to confirm differential expression.

For the purpose of gene profiling experiments in separated CD8^+^ T-cell subpopulations, unprocessed peripheral blood samples were stained with the following fluorochrome-conjugated mouse anti-human monoclonal antibodies: anti-CD8a APC-eFluor 780 (eBioscience), anti-CD45RA APC (eBioscience) and anti-CCR7 FITC (R&D System). After red blood cell lysis, the subpopulations of CD8^+^ T-lymphocytes were flow sorted in a FACS Aria (BD) instrument according to the gating strategy previously described [10] as: naïve (T_N_, CD8^+^CCR7^+^CD45RA^+^), central memory (T_CM_, CD8^+^CCR7^+^CD45RA^-^) and effector memory (T_EM_, CD8^+^CCR7^-^CD45RA^+/-^). Samples were sorted until 3000 cells from each gated subpopulation were collected. Cells were sorted according to the gating strategy illustrated in Fig 1A. In order to avoid the risk of low representativity after cell sorting, we did not further discriminate effector memory cells according to CD45RA expression as previously described [10]. This decision was supported by our previous observation that the cell subpopulation that mostly contributes to the variation in the total number of peripheral CD8^+^ T cells, both in HH patients and normal controls, is the entire effector memory population [10]. Total RNA was isolated from sorted cells using the RNeasy Plus Micro Kit (QIAGEN), according to the manufacturer’s guidelines. Gene expression in sorted CD8^+^ T-cell subpopulations: was assessed for a group of specific candidate genes: *LEF1*, *ACTN1*, *CCR7*; *NAA50*; *P2RY8* and *FOSL2*. cDNAs resulting from the reverse transcription reaction with SuperScript First-Strand Synthesis System (Invitrogen) were subjected to a first round of PCR amplification with specific primers (S6 Table). To quantify the expression levels of all genes of interest in each CD8^+^ T-lymphocyte subpopulation, a second seminested real-time PCR was performed in an iCycler iQ5 (Bio-Rad) using iQ SYBR Green Supermix (Bio-Rad) [53]. At the end of the PCR cycling, melting curves were generated to ascertain the amplification of a single product and the absence of primer dimers. Results were normalized to GAPDH as endogenous control.

#### b) Apoptosis and cell cycle studies in HH patients

Twenty HH patients and 12 controls were used for apoptosis and cell cycle studies. These included 10 patients classified with the low CD8 phenotype and 10 patients classified as normal/high CD8 phenotype, as described above. During the study period, patients were evaluated at different stages of their treatment course. Four patients were under intensive phlebotomy treatment and the remaining 16 were receiving maintenance therapy. The inclusion of patients at different stages of iron load was also important in order to allow an analysis of cell cycle parameters in relation to a wide range of transferrin saturation values.

For detection of apoptotic T cells, blood samples were collected into tubes containing sodium heparin and PBMCs were separated by centrifugation on the Lymphoprep 1.077g/ml density gradient (Axis-Shield) after 12 hour resting at 4°C. Apoptotic T cells from HH patients were assessed through flow cytometry, using the Annexin V-FITC/7-AAD Kit (Beckman Coulter, BC), containing FITC conjugated Annexin V, 7-AAD staining solution and Annexin V binding buffer, following the manufactures’ instructions. Samples were acquired in a flow cytometer EPICS-XL-MCL (BC) using the System II software (BC). Data were analyzed using the System II software (BC). The percentage of viable (Annexin V negative/7-AAD negative), early apoptotic (Annexin V positive/7-AAD negative) and late apoptotic or already dead (Annexin V positive/7-AAD positive) CD8^+^ and CD4^+^ T cells was calculated.

In order to analyze the distribution of CD8^+^ and CD4^+^ T cells throughout the cell cycle phases: blood sample was submitted to cell surface immunophenotyping with FITC-conjugated mouse anti-human CD4 or anti-human CD8 IgG mAb followed by staining with FITC conjugated rabbit anti-mouse IgG polyclonal Ab and cellular DNA measurement using the DNA PREP Reagents Kit (BC) according to a protocol that was described in detail elsewhere [54]. After staining, samples were acquired in an EPICS-XL-MCL flow cytometer (BC) using the System II software (BC). Data were analyzed using specific software for DNA analysis Multicyle for Windows (Phoenix Flow System, PFS). The percentages of CD8^+^ and CD4^+^ T cells in each cell cycle phase (G0/G1, S and G2/M) were calculated.

#### c) Genome-wide expression analysis of CD8^+^ T lymphocytes from Hfe-/- and wild type mice

Peripheral blood from each mice (n = 16) was collected by heart puncture (approximately 500μl) and CD8^+^ T-lymphocyte population was isolated by FACS-ARIA I cell sorting using anti-CD8 APC antibody (BD Bioscience). The purities of isolated CD8^+^ T cells were measured by flow-cytometric analysis of cell markers (CD8) and in all samples >95% of purity was obtained. The maximum number of CD8^+^ T cells was sorted by each mouse blood sample. Total RNA was extracted with RNeasy Plus Micro kit (QIAGEN) according to the manufacture`s guidelines. Total RNA integrity was evaluated by Agilent 2010 Bioanalyzer protocol. To overcome the low RNA concentration, we performed the Ovation Pico WTA system protocol by NuGEN to amplify the RNA samples followed by cDNA synthesis using the WT Ovation Exon module.

Transcriptional profile of CD8^+^ T lymphocytes from mice was assessed using the GeneChip Mouse Gene ST 1.0 array (Affymetrix, Santa Clara, CA, USA). The RNA processing and hybridization steps, carried out at the Genomics Core facility of the European Molecular Biology Laboratory (EMBL, Heidelberg), were performed as recommended by the manufacturer. Data from the GeneChip were imported into GeneSpring GX 11.5 software (Agilent) and the expression value for each gene was normalized by using the Robust Multichip Average (RMA) 16 algorithm. Results were grouped according to mice genotype and iron diet conditions, i.e. wild type mice with normal or high-iron diet, and *Hfe* ^-/-^ with normal or high iron diet. Genes which exhibited at least a 1.8-fold change in expression were considered as up- or down-regulated.

#### d) Human translational study of the candidate genes found differently expressed in Hfe^-/-^ mice

Subsequent studies were done for the most significantly different expressed genes found in mice study, calgranulin genes *S100a8* and *S100a9*, consisting in mRNA and protein expression in HH patients and controls.

Gene expression of *S100a8* and *S100a9* was assessed in sorted CD8^+^ T cells from HH patients and controls. For this, PBMCs were isolated from whole blood or buffy coat samples by density separation over Lymphoprep and CD8^+^ T-lymphocytes were selected MACS, as described above. Total RNA, of MACS-purified CD8^+^ T cells, was extracted as described above. In each experiment time total RNA concentration was normalized between patients and controls and converted into cDNA with NZY First-Strand cDNA Synthesis kit (Nzytech) as recommended by the manufacturer. Quantitative real-time PCR was performed in an iCycler iQ5 (Bio-Rad) using iQ SYBR Green Supermix (Bio^-^Rad) using specific primers for each gene (S7 Table). At the end of the PCR cycling, melting curves were generated to ascertain the amplification of a single product and the absence of primer dimers. Results were normalized to 18S gene as endogenous control. In order to access the reproducibility of the technique, biological replicates were performed consisting in the analysis of different samples of the same patient in different days. This was always done against a different control for the experiment of the day (n = 12). Since reproducibility of the replicates was achieved, all samples were included for analysis (n = 12). Paired analysis of patient-control of the day was performed.

Intracellular protein expression of S100a9 was accessed by flow cytometry in total peripheral blood samples from 30 HH patients and 13 controls. For this, peripheral blood was collected into K3-ethylene-diamine-tetracetic acid (EDTA-K3)-containing tubes. The blood samples were stained with the following fluorochrome-conjugated mouse anti-human monoclonal antibodies: anti-human CD3 FITC (eBioscience) and anti-human CD8 PerCP (eBioscience). Leukocyte fixation and subsequent permeabilization of the cells was then performed using the Fix & Perm Cell Permeabilization Kit (Invitrogen). Anti-human IgG1 S100a9 PE (sc-53187, Santa Cruz, CA) or the PE-conjugated isotype control was used for intracellular staining. Samples were acquired in FACS Canto v.2 flow cytometer (BD) under the same conditions, using the FACSDiva software (BD). In each experiment day at least a control sample was tested in parallel with patient’s samples. Data were analyzed in the Infinicyt software (Cytognos SL, Salamanca, Spain) and the median fluorescence intensity (MFI) of the S100a9 expression was determined in neutrophils, monocytes and lymphocytes defined according to SSC and FCS characteristics, in CD8^+^ lymphocytes defined by the CD3^+^CD8^+^ population after lymphocyte gating and in CD4^+^ lymphocytes defined as CD3^+^CD8^-^ population after lymphocyte gating. Results were analyzed as a ratio of patient/control of the day.

### Statistical analysis

Correlations among variables were analyzed by multiple-variable correlation analysis with calculation of various statistics, including covariances and partial correlations. Differences in group means or sample distributions were tested respectively by the Student’s t-test and the Kolmogorov-Smirnov (KS) two sample test as appropriate. For analysis of Affymetrix expression data we used the normalized values. For analysis of individual expression values in sorted CD8^+^ T-cell subsets, a systematic outliers’ exclusion was performed in HH patients´ data in order to permit comparisons with controls’ group means assuming equal variances. The non-parametric Wilcoxon test for paired samples was used to compare mRNA expression levels of *S100a8* and *S100a9* between patients and controls analyzed on the same day. The parametric paired T-test was used to compare protein expression of S100a9 by FACS (MFI) between patients and controls analyzed in the same day.

Analysis of differentially expressed genes, resulting from genome-wide studies in mice, was clustered into biologically relevant categories using the bioinformatics resources: Database for Annotation, Visualization and Integrated Discovery (DAVID, http://david.abcc.ncifcrf.gov/) and Web-based Gene SeT AnaLysis Toolkit (WebGestalt, http://bioinfo.vanderbilt.edu/wg2/). For Gene Ontology (GO) and Kyoto Encyclopedia of Genes and Genomes (KEGG) pathways enrichment analysis, the minimum of two genes were required for identification of relevant biological pathway, with statistically significance. The *p* value of 0.05 was taken as the level of statistical significance. Affymetrix data were analyzed in GeneSpring GX software (Agilent) and all other statistical analysis were performed with StatGraphics software (Statgraphics Statistical Graphics System, version 16.0).

## Supporting Information

S1 Fig Positive (A) and negative (B) correlations of *S100a8* and *S100a9* with the other differentially expressed genes. Results were obtained by multi-variable correlation analysis of the normalized gene expression values in individual mice on high iron diet conditions. The partial correlation coefficients and significance levels (*P value in brackets*) for the different gene combinations are shown. The relative strength of the correlations is highlighted by colour grading of blue (for positive correlations) or yellow (for negative correlation). Genes are ordered by the strength of their associations with *S100a9*.
(TIF)

S1 Table Clinical correlations of the genes identified in the genome-wide differential screen. (PDF)

S2 Table Genes up and down regulated in *Hfe* knockout CD8^+^ T lymphocytes in comparison with C57BL/6 mice under normal diet condition. (PDF)

S3 Table Genes up and down regulated in *Hfe* knockout CD8^+^ T lymphocytes in comparison with C57BL/6 mice under high iron diet condition. (PDF)

S4 Table Functional categories of the significantly different expressed genes between *Hfe* knockout and wild type. (PDF)

S5 Table Expression levels of iron related genes in CD8^+^ T lymphocytes from *Hfe* knockout and wild type C57BL/6 mice The relative levels of expression are highlighted by colour grading according to normalized expression values. The significance of the differential expression values is indicated by the *p* value of T-test.
(PDF)

S6 Table Sequences of the oligonucleotide primers used for the first and second PCR experiments in CD8^+^ T lymphocytes from HH patients. (PDF)

S7 Table Sequences of the oligonucleotide primers used for S100a8 and S100a9 expression studies in sorted CD8 from HH patients and controls. (PDF)
