# Supplementary figures and images for: Lymphocyte Gene Expression Signatures from Patients and Mouse Models of Hereditary Hemochromatosis Reveal a Function of HFE as a Negative Regulator of CD8+ T-Lymphocyte Activation and Differentiation In Vivo
Source: PLoS One. 2015 Apr 16;10(4):e0124246. doi: 10.1371/journal.pone.0124246 (PMC4399836; doi:10.1371/journal.pone.0124246)

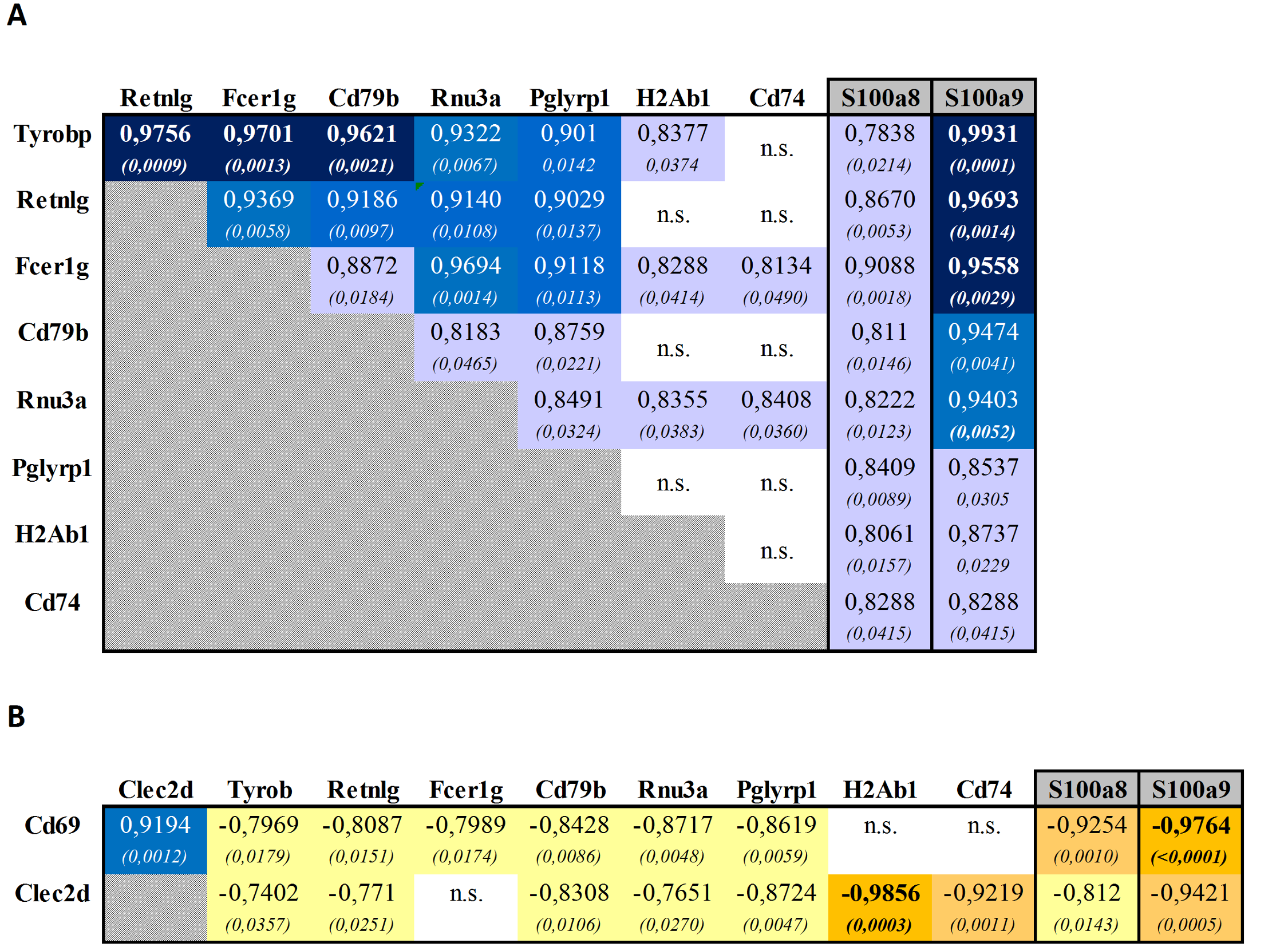

Supplement: S1 Fig — Results were obtained by multi-variable correlation analysis of the normalized gene expression values in individual mice on high iron diet conditions. The partial correlation coefficients and significance levels (P value in brackets) for the different gene combinations are shown. The relative strength of the correlations is highlighted by colour grading of blue (for positive correlations) or yellow (for negative correlation). Genes are ordered by the strength of their associations with S100a9. (TIF) [file pone.0124246.s001.TIF]
