# Supplementary material for: Lymphocyte Gene Expression Signatures from Patients and Mouse Models of Hereditary Hemochromatosis Reveal a Function of HFE as a Negative Regulator of CD8+ T-Lymphocyte Activation and Differentiation In Vivo
Source: PLoS One. 2015 Apr 16;10(4):e0124246. doi: 10.1371/journal.pone.0124246 (PMC4399836; doi:10.1371/journal.pone.0124246)
Supplement: S1 Table — (PDF) [file pone.0124246.s002.pdf]

S1 Table: **Clinical correlations of the genes identified in the genome-wide differential screen**. Indicated are the correlation coefficient (r) R squared (R^2) and significance level (p) values obtained by simple regression analyses between the mRNA expression levels for each indicated gene and the clinical variables reflecting the immune-phenotype (CD8 T lymphocyte and subset counts) and the iron overload profile defined by estimated total body iron stores (TBIS). For each clinical variable are indicated the ranges of values in the total HH patient population analysed.

mRNA expression levels		CD8 T cell subsets		
	**Total CD8 T lymphocytes (0,09-0,73x10^6/ml)**	**naive + central memory (0,044-0,459x10^6/ml)**	**effector memory (0,060-0,403x10^6/ml)**	**TBIS (1,5-17,4g)**
**CCR7**	*n.s.*	*r=+0,95 R^2=91% (p=0,0032)*	*n.s.*	*r=-0,78 R^2=61% (p=0,0129)*
**LEF1**	*n.s.*	*r=+0,79 R^2=63% (p=0,0601)*	*n.s.*	*r=-0,70 R^2=48% (p=0,0379)*
**ACTN1**	*n.s.*	*r=+0,87 R^2=75% (p=0,0249)*	*n.s.*	*r=-0,79 R^2=62% (p=0,0115)*
**NAT13**	*r=-0,79 R^2=62% (p=0,0070)*	*n.s.*	*r=-0,91 R^2=84% (p=0,0108)*	*n.s.*
**P2YR8**	*r=-0,86 R^2=74% (p=0,0015)*	*n.s.*	*r=-0,88 R^2=78% (p=0,0191)*	*n.s.*
**FOSL2**	*r=-0,70 R^2=49% (p=0,0243)*	*n.s.*	*r=-0,81 R^2=65% (p=0,0525)*	*n.s.*

n.s.=not statistically significant
