# Supplementary material for: Lymphocyte Gene Expression Signatures from Patients and Mouse Models of Hereditary Hemochromatosis Reveal a Function of HFE as a Negative Regulator of CD8+ T-Lymphocyte Activation and Differentiation In Vivo
Source: PLoS One. 2015 Apr 16;10(4):e0124246. doi: 10.1371/journal.pone.0124246 (PMC4399836; doi:10.1371/journal.pone.0124246)
Supplement: S2 Table — (PDF) [file pone.0124246.s003.pdf]

**S2 Table Genes up and down regulated in *Hfe* knockout $CD8^+$ T lymphocytes in comparison with C57BL/6 mice under normal diet condition**

| Transcripts ID | Gene abbreviation | Gene name | Fold Change | *p*-value | Gene ID |
|---|---|---|---|---|---|
| **10493831** | S100a8 | S100 calcium binding protein A8 (calgranulin A) | 16,53 | 0,00 | 20201 |
| **10499861** | S100a9 | S100 calcium binding protein A9 (calgranulin B) | 12,96 | 0,00 | 20202 |
| **10362674** | Rnu3a | U3A small nuclear RNA | 5,31 | 0,00 | 19850 |
| **10436100** | Retnlg | resistin like gamma | 4,67 | 0,00 | 245195 |
| **10456005** | Cd74 | CD74 antigen (invariant polypeptide of major histocompatibility complex, class II antigen-associated) | 4,62 | 0,01 | 16149 |
| **10372648** | Lyz2 | lysozyme 2 | 4,33 | 0,01 | 17105 |
| **10444291** | H2-Ab1 | histocompatibility 2, class II antigen A, beta 1 | 3,75 | 0,01 | 14961 |
| **10493812** | S100a4 | S100 calcium binding protein A4 | 3,44 | 0,00 | 20198 |
| **10450154** | H2-Aa | histocompatibility 2, class II antigen A, alpha | 3,35 | 0,01 | 14960 |
| **10570434** | Ifitm1 | interferon induced transmembrane protein 1 | 3,12 | 0,00 | 68713 |
| **10551025** | Cd79a | CD79A antigen (immunoglobulin-associated alpha) | 2,97 | 0,00 | 12518 |
| **10468517** | Mxi1 | Max interacting protein 1 | 2,90 | 0,03 | 17859 |
| **10512487** | Rmrp | RNA component of mitochondrial RNAase P | 2,81 | 0,02 | 19782 |
| **10444236** | H2-DMb1/DMb2 | histocompatibility 2, class II, locus Mb1 and Mb2 | 2,76 | 0,00 | 15000 \| 14999 |
| **10508465** | Marcksl1 | MARCKS-like 1 | 2,68 | 0,00 | 17357 |
| **10551883** | Tyrobp | TYRO protein tyrosine kinase binding protein | 2,63 | 0,02 | 22177 |
| **10508721** | Snora44 | small nucleolar RNA, H/ACA box 44 | 2,59 | 0,03 | 100217418 |
| **10550509** | Pglyrp1 | peptidoglycan recognition protein 1 | 2,50 | 0,00 | 21946 |
| **10360070** | Fcer1g | Fc receptor, IgE, high affinity I, gamma polypeptide | 2,49 | 0,04 | 14127 |
| **10467979** | Scd1 | stearoyl-Coenzyme A desaturase 1 | 2,47 | 0,03 | 20249 |
| **10429520** | Ly6d | lymphocyte antigen 6 complex, locus D | 2,40 | 0,03 | 17068 |
| **10538871** | Gm4964 | predicted gene 4964 | 2,37 | 0,04 | 243420 |
| **10440576** | Rnf160 | ring finger protein 160 | 2,36 | 0,00 | 78913 |
| **10392142** | Cd79b | CD79B antigen | 2,20 | 0,01 | 15985 |
| **10558769** | Ifitm1 | interferon induced transmembrane protein 1 | 2,19 | 0,01 | 68713 |
| **10379727** | Gm11428 | predicted gene 11428 | 2,10 | 0,03 | 100034251 |
| **10414262** | Ear2 | eosinophil-associated, ribonuclease A family, member 2 | 2,08 | 0,03 | 13587 |
| **10349593** | Faim3 | Fas apoptotic inhibitory molecule 3 | 2,06 | 0,00 | 69169 |
| **10548817** | Plbd1 | phospholipase B domain containing 1 | 2,04 | 0,05 | 66857 |
| **10466172** | Ms4a1 | membrane-spanning 4-domains, subfamily A, member 1 | 1,99 | 0,05 | 12482 |
| **10548535** | Klra3 | killer cell lectin-like receptor, subfamily A, member 3 | 1,97 | 0,03 | 16634 |
| **10430818** | Tnfrsf13c | tumor necrosis factor receptor superfamily, member 13c | 1,97 | 0,01 | 72049 |
| **10556113** | Rbm3 | RNA binding motif protein 3 | 1,95 | 0,00 | 19652 |
| **10481627** | Lcn2 | lipocalin 2 | 1,91 | 0,00 | 16819 |
| **10535458** | Zdhhc4 | zinc finger, DHHC domain containing 4 | 1,89 | 0,03 | 72881 |
| **10563338** | Ppp1r15a | protein phosphatase 1, regulatory (inhibitor) subunit 15A | 1,87 | 0,03 | 17872 |
| **10422227** | Spry2 | sprouty homolog 2 (Drosophila) | 1,86 | 0,04 | 24064 |
| **10398286** | Mir342 | microRNA 342 | -4,69 | 0,01 | 723909 |
| **10515694** | Szt2 | seizure threshold 2 | -3,04 | 0,02 | 230676 |
| **10503198** | Chd7 | chromodomain helicase DNA binding protein 7 | -3,02 | 0,01 | 320790 |
| **10405779** | Mir23b | microRNA 23b | -2,78 | 0,03 | 387217 |
| **10456490** | Cep192 | centrosomal protein 192 | -2,66 | 0,02 | 70799 |
| **10512827** | Gm568 | predicted gene 568 | -2,50 | 0,03 | 230143 |
| **10351043** | Snord47 | small nucleolar RNA, C/D box 47 | -2,50 | 0,02 | 100217446 |
| **10410311** | Zfp456 | zinc finger protein 456 | -2,43 | 0,02 | 408065 |
| **10447036** | n-R5s65 | nuclear encoded rRNA 5S 65 | -2,39 | 0,04 | |
| **10523134** | Pf4 | platelet factor 4 | -2,35 | 0,04 | 56744 |
| **10503218** | Chd7 | chromodomain helicase DNA binding protein 7 | -2,34 | 0,05 | 320790 |
| **10434396** | Abcf3 | ATP-binding cassette, sub-family F (GCN20), member 3 | -2,29 | 0,00 | 27406 |
| **10412900** | Nkiras1 | NFKB inhibitor interacting Ras-like protein 1 | -2,21 | 0,00 | 69721 |
| **10502934** | Rabggtb | RAB geranylgeranyl transferase, b subunit | -2,21 | 0,00 | 19352 |
| **10531776** | Fam175a | family with sequence similarity 175, member A | -2,10 | 0,00 | 70681 |
| **10548333** | Cd69 | CD69 antigen | -2,08 | 0,00 | 12515 |
| **10457838** | Zfp397os | zinc finger protein 397 opposite strand | -2,04 | 0,00 | 328918 |
| **10586250** | Dennd4a | DENN/MADD domain containing 4A | -2,03 | 0,02 | 102442 |

| | | | | | |
|---|---|---|---|---|---|
| **10546706** | Rybp | RING1 and YY1 binding protein | -2,01 | 0,02 | 56353 |
| **10574141** | Nlrc5 | NLR family, CARD domain containing 5 | -1,99 | 0,04 | 434341 |
| **10442032** | BC002059 | cDNA sequence BC002059, mRNA (cDNA clone MGC:6110 ) | -1,90 | 0,01 | 213811 |
| **10410530** | Slc6a19 | solute carrier family 6 (neurotransmitter transporter), member 19 | -1,90 | 0,04 | 74338 |
| **10542156** | Clec2d | C-type lectin domain family 2, member d | -1,89 | 0,00 | 93694 |
| **10525487** | 4932422M17Rik | RIKEN cDNA 4932422M17 gene | -1,89 | 0,03 | 74366 |
| **10522009** | Pgm1 | phosphoglucomutase 1 | -1,87 | 0,02 | 66681 |
| **10515363** | Mmachc | methylmalonic aciduria cblC type, with homocystinuria | -1,87 | 0,05 | 67096 |
| **10443459** | Sfrs3 | splicing factor, arginine/serine-rich 3 (SRp20) | -1,85 | 0,00 | 20383 |
| **10492582** | Mir15b | microRNA 15b | -1,85 | 0,05 | 387175 |
| **10501048** | Dennd2d | DENN/MADD domain containing 2D | -1,84 | 0,02 | 72121 |
