# Supplementary material for: Lymphocyte Gene Expression Signatures from Patients and Mouse Models of Hereditary Hemochromatosis Reveal a Function of HFE as a Negative Regulator of CD8+ T-Lymphocyte Activation and Differentiation In Vivo
Source: PLoS One. 2015 Apr 16;10(4):e0124246. doi: 10.1371/journal.pone.0124246 (PMC4399836; doi:10.1371/journal.pone.0124246)
Supplement: S3 Table — (PDF) [file pone.0124246.s004.pdf]

S3 Table: Genes up and down regulated in *Hfe* knockout CD8$^+$ T lymphocytes in comparison with C57BL/6 mice under high iron diet condition

Transcripts ID	Gene abbreviation	Gene name	Fold Change	*p*-value	Gene ID
**10493831**	S100a8	S100 calcium binding protein A8 (calgranulin A)	34,58	0,00	20201
**10499861**	S100a9	S100 calcium binding protein A9 (calgranulin B)	29,21	0,00	20202
**10362674**	Rnu3a	U3A small nuclear RNA	9,10	0,00	19850
**10435497**	Stfa2l1	stefin A2 like 1	6,49	0,05	268885
**10436100**	Retnlg	resistin like gamma	6,36	0,00	245195
**10456005**	Cd74	CD74 antigen (invariant polypeptide of major histocompatibility complex, class II antigen-associated)	3,71	0,05	16149
**10551883**	Tyrobp	TYRO protein tyrosine kinase binding protein	3,48	0,00	22177
**10399428**	Snord118	small nucleolar RNA, C/D box 118	3,25	0,00	100216530
**10377429**	Snord118	small nucleolar RNA, C/D box 118	3,25	0,00	100216530
**10383756**	Ifitm2	interferon induced transmembrane protein 2	3,18	0,01	80876
**10569017**	Ifitm3	interferon induced transmembrane protein 3	3,18	0,03	66141
**10379727**	Gm11428	predicted gene 11428	2,89	0,02	100034251
**10444291**	H2-Ab1	histocompatibility 2, class II antigen A, beta 1	2,77	0,03	14961
**10551025**	Cd79a	CD79A antigen (immunoglobulin-associated alpha)	2,74	0,04	12518
**10550509**	Pglyrp1	peptidoglycan recognition protein 1	2,65	0,01	21946
**10539577**	Spr	sepiapterin reductase	2,60	0,04	20751
**10583286**	Gpr83	G protein-coupled receptor 83	2,58	0,04	14608
**10545014**	Vopp1	vesicular, overexpressed in cancer, prosurvival protein 1	2,51	0,03	232023
**10548333**	Cd69	CD69 antigen	2,49	0,00	12515
**10360070**	Fcer1g	Fc receptor, IgE, high affinity I, gamma polypeptide	2,46	0,00	14127
**10427908**	Gm9948	predicted gene 9948	2,45	0,02	791293
**10553299**	Ifitm2	interferon induced transmembrane protein 2	2,42	0,02	80876
**10344799**	Cspp1	centrosome and spindle pole associated protein 1	2,42	0,02	211660
**10527638**	Alox5ap	arachidonate 5-lipoxygenase activating protein	2,40	0,01	11690
**10368508**	2610036L11Rik	RIKEN cDNA 2610036L11 gene	2,38	0,02	66311
**10397536**	Gm4005	predicted gene 4005	2,37	0,02	100042747
**10588223**	Anapc13	anaphase promoting complex subunit 13	2,36	0,01	69010
**10493820**	S100a6	S100 calcium binding protein A6 (calcyclin)	2,36	0,00	20200
**10364293**	Ube2g2	ubiquitin-conjugating enzyme E2G 2	2,28	0,04	22213
**10473250**	Mrpl18	mitochondrial ribosomal protein L18	2,21	0,02	67681
**10569014**	Ifitm2	interferon induced transmembrane protein 2	2,21	0,01	80876
**10437963**	Fam128b	family with sequence similarity 128, member B	2,19	0,00	72083
**10590298**	Eif1b	eukaryotic translation initiation factor 1B	2,18	0,01	68969
**10452110**	2410015M20Rik	RIKEN cDNA 2410015M20 gene	2,16	0,01	224904
**10603833**	Usmg5	upregulated during skeletal muscle growth 5	2,14	0,02	66477
**10542156**	Clec2d	C-type lectin domain family 2, member d	2,14	0,01	93694
**10392142**	Cd79b	CD79B antigen	2,12	0,01	15985
**10412211**	Gzma	granzyme A	2,11	0,00	14938
**10514466**	Jun	jun proto-onogene	2,11	0,01	16476
**10575961**	Usp10	ubiquitin specific peptidase 10	2,10	0,01	22224
**10356999**	Prdx2	peroxiredoxin 2	2,09	0,04	21672
**10517336**	Clic4	chloride intracellular channel 4 (mitochondrial)	2,08	0,02	29876
**10440918**	Tmem50b	transmembrane protein 50B	2,07	0,04	77975
**10362896**	Cd24a	CD24a antigen	2,05	0,01	12484
**10565081**	Timm17a	translocase of inner mitochondrial membrane 17a	2,03	0,00	21854
**10576391**	Rab4a	RAB4A, member RAS oncogene family	1,99	0,03	19341
**10416950**	Mir18	microRNA 18	1,98	0,03	387135
**10605943**	Pdzd11|Kif4	PDZ domain containing 11 | kinesin family member 4	1,97	0,01	72621 | 16571
**10604019**	1810037I17Rik	RIKEN cDNA 1810037I17 gene	1,97	0,05	67704
**10574151**	Nlrc5	NLR family, CARD domain containing 5	1,96	0,02	434341
**10396862**	Actn1|Strm	actinin, alpha 1 | striamin	1,93	0,01	109711
**10468287**	Usmg5	upregulated during skeletal muscle growth 5	1,92	0,03	66477
**10414958**	Tcra-V8	T-cell receptor alpha, variable 8	1,92	0,04	100043322
**10450814**	Ppp1r11	protein phosphatase 1, regulatory (inhibitor) subunit 11	1,91	0,05	76497
**10487476**	1500011K16Rik	RIKEN cDNA 1500011K16 gene	1,91	0,01	67885

**10490221**	Atp5e	ATP synthase, H+ transporting, mitochondrial F1 complex, epsilon subunit	1,91	0,02	67126
**10371002**	Lsm7	LSM7 homolog, U6 small nuclear RNA associated (S. cerevisiae)	1,90	0,05	66094
**10430778**	Phf5a	PHD finger protein 5A	1,90	0,03	68479
**10345183**	Cdk10	cyclin-dependent kinase 10	1,89	0,01	234854
**10349648**	Ctse	cathepsin E	1,88	0,04	13034
**10603837**	Ndufb11	NADH dehydrogenase (ubiquinone) 1 beta subcomplex, 11	1,87	0,02	104130
**10492757**	Plrg1	pleiotropic regulator 1, PRL1 homolog (Arabidopsis)	1,87	0,02	53317
**10416199**	Entpd4	ectonucleoside triphosphate diphosphohydrolase 4	1,86	0,04	67464
**10512901**	Mrpl50	mitochondrial ribosomal protein L50	1,85	0,00	28028
**10450640**	Mrps18b	mitochondrial ribosomal protein S18B	1,85	0,00	66973
**10449356**	AI413582	expressed sequence AI413582	1,83	0,03	106672
**10373577**	Ormdl2|Dnajc14	ORM1-like 2 (S. cerevisiae) | DnaJ (Hsp40) homolog, subfamily C, member 14	1,83	0,02	66844 | 74330
**10366043**	Dusp6	dual specificity phosphatase 6	1,81	0,04	67603
**10548333**	Cd69	CD69 antigen	-2,49	0,00	12515
**10396862**	Actn1|Strm	actinin, alpha 1 | striamin	-1,93	0,01	109711 | 20904
**10542156**	Clec2d	C-type lectin domain family 2, member d	-2,14	0,01	93694
**10345183**	Cdk10	cyclin-dependent kinase 10	-1,89	0,01	234854
**10575961**	Usp10	ubiquitin specific peptidase 10	-2,10	0,01	22224
**10574151**	Nlrc5	NLR family, CARD domain containing 5	-1,96	0,02	434341
**10344799**	Cspp1	centrosome and spindle pole associated protein 1	-2,42	0,02	211660
**10397536**	Gm4005	predicted gene 4005	-2,37	0,02	100042747
**10583286**	Gpr83	G protein-coupled receptor 83	-2,58	0,04	14608
**10416950**	Mir18	microRNA 18	-1,98	0,03	387135
**10416199**	Entpd4	ectonucleoside triphosphate diphosphohydrolase 4	-1,86	0,04	67464
