# Supplementary material for: Lymphocyte Gene Expression Signatures from Patients and Mouse Models of Hereditary Hemochromatosis Reveal a Function of HFE as a Negative Regulator of CD8+ T-Lymphocyte Activation and Differentiation In Vivo
Source: PLoS One. 2015 Apr 16;10(4):e0124246. doi: 10.1371/journal.pone.0124246 (PMC4399836; doi:10.1371/journal.pone.0124246)
Supplement: S4 Table — (PDF) [file pone.0124246.s005.pdf]

S4 Table: Functional categories of the significantly different expressed genes between *Hfe* knockout and wild type

Category	Term	Count	*P* value	Genes
**Cluster 1**				
SP_PIR_KEYWORDS	Immune response	8	3.41E-07	Cd79a, Cd74, H2-Ab1, H2-Aa, Pglyrp1, Tnfrsf13c, Cd79b, Faim3
GOTERM_BP_FAT	Antigen processing and presentation of exogenous peptide antigen	5	9.42E-07	Cd74, H2-DMb1, Fcer1g, H2-Ab1, H2-Aa
GOTERM_BP_FAT	Positive regulation of leukocyte activation	4	1.95E-03	Cd74, Fcer1g, H2-Aa, Tnfrsf13c
**Cluster 2**				
GOTERM_CC_FAT	Late endosome	4	2.18E-04	Cd79a, Cd74, H2-DMb1, H2-Ab1
KEGG_PATHWAY	Intestinal immune network for IgA production	4	4.92E-04	H2-DMb1, H2-Ab1, H2-Aa, Tnfrsf13c
KEGG_PATHWAY	Graft-versus-host disease	4	5.85E-04	Klra3, H2-DMb1, H2-Ab1, H2-Aa
**Cluster 3**				
GOTERM_BP_FAT	Positive regulation of response to stimulus	6	1.02E-04	Cd79a, Fam175a, Fcer1g, H2-Aa, Tnfrsf13c, Cd79b
INTERPRO	Phosphorylated immunoreceptor signaling ITAM	3	1.31E-04	Cd79a, Fcer1g, Cd79b
GOTERM_BP_FAT	Immune response-activating signal transduction	3	6.86E-03	Cd79a, Fcer1g, Cd79b
GOTERM_BP_FAT	Immune response-regulating signal transduction	3	8.04E-03	Cd79a, Fcer1g, Cd79b
**Cluster 4**				
GOTERM_BP_FAT	Immune response	11	1.83E-07	Pf4, Cd79a, Cd74, H2-DMb1, Fcer1g, H2-Ab1, H2-Aa, Pglyrp1, Tnfrsf13c, Cd79b, Faim3
SP_PIR_KEYWORDS	disulfide bond	17	1.50E-04	Lcn2, Pf4, Klra3, Tyrobp, Cd79a, Lyz2, H2-Ab1, H2-Aa, Pglyrp1, Tnfrsf13c, Cd79b, Faim3, Cd74, Fcer1g, Ly6d, Clec2d, Cd69
SP_PIR_KEYWORDS	signal	16	3.59E-03	Lcn2, Pf4, Tyrobp, Cd79a, H2-DMb1, Lyz2, H2-Ab1, H2-Aa, Pglyrp1, Cd79b, Faim3, Retnlg, Gm11428, Fcer1g, Ly6d, Plbd1
INTERPRO	IPR007110:Immunoglobulin-like	5	3.44E-02	Cd79a, H2-DMb1, H2-Ab1, H2-Aa, Cd79b
**Cluster 5**				
GOTERM_BP_FAT	Positive regulation of immune system process	6	1.67E-04	Cd79a, Cd74, Fcer1g, H2-Aa, Tnfrsf13c, Cd79b
GOTERM_BP_FAT	GO:0001775~cell activation	5	3.60E-03	Ms4a1, Pf4, Cd79a, Cd74, Fcer1g
GOTERM_BP_FAT	GO:0045321~leukocyte activation	4	1.86E-02	Ms4a1, Cd79a, Cd74, Fcer1g
**Cluster 6**				
UP_SEQ_FEATURE	calcium-binding region:2; high affinity	3	4.23E-04	S100a8, S100a4, S100a9
UP_SEQ_FEATURE	calcium-binding region:1; low affinity	3	4.23E-04	S100a8, S100a4, S100a9
PIR_SUPERFAMILY	PIRSF002353:S-100 protein	3	4.73E-04	S100a8, S100a4, S100a9
INTERPRO	IPR001751:S100/CaBP-9k-type, calcium binding	3	1.54E-03	S100a8, S100a4, S100a9
SP_PIR_KEYWORDS	EF hand	3	2.22E-03	S100a8, S100a4, S100a9
SP_PIR_KEYWORDS	calcium binding	3	6.17E-03	S100a8, S100a4, S100a9
**Cluster 7**				
GOTERM_BP_FAT	Leukocyte chemotaxis	3	2.30E-03	Pf4, Fcer1g, S100a9
GOTERM_BP_FAT	Cell chemotaxis	3	2.30E-03	Pf4, Fcer1g, S100a9
GOTERM_BP_FAT	Chemotaxis	4	2.8E-03	Pf4, S100a8, Fcer1g, S100a9
GOTERM_BP_FAT	Locomotory behavior	5	3.09E-03	Pf4, S100a8, Chd7, Fcer1g, S100a9
**Cluster 8**				
GOTERM_BP_FAT	Defense response	6	5.11E-03	Cd74, Fcer1g, Lyz2, H2-Aa, Pglyrp1, Clec2d
GOTERM_BP_FAT	Defense response to bacterium	3	3.04E-02	Fcer1g, Lyz2, Pglyrp1
**Cluster 9**				
GOTERM_MF_FAT	Carbohydrate binding	5	7.02E-03	Pf4, Klra3, Pglyrp1, Clec2d, Cd69
SP_PIR_KEYWORDS	Signal-anchor	5	1.81E-02	Klra3, Cd74, Tnfrsf13c, Clec2d, Cd69
UP_SEQ_FEATURE	domain:C-type lectin	3	2.21E-02	Klra3, Clec2d, Cd69
INTERPRO	IPR018378:C-type lectin, conserved site	3	2.82E-02	Klra3, Clec2d, Cd69
INTERPRO	IPR001304:C-type lectin	3	3.50E-02	Klra3, Clec2d, Cd69
INTERPRO	IPR016186:C-type lectin-like	3	4.01E-02	Klra3, Clec2d, Cd69
SMART	SM00034:CLECT	3	4.16E-02	Klra3, Clec2d, Cd69

The Category columm shows the original database/resource from which terms originate. The Term columm indicates the enriched terms associated with the gene list. The Count columm indicates the number of genes involved in the term. The *P* value was obtained with the modified Fisher exact *test*. BP, biological processes; GO, Gene Ontology Term; KEGG, Kyoto Encyclopedia of Genes and Genomes; MF, molecular functions; PIR, Protein Information Resource; UDP, Uridine 5'-diphospho
