# Supplementary material for: Lymphocyte Gene Expression Signatures from Patients and Mouse Models of Hereditary Hemochromatosis Reveal a Function of HFE as a Negative Regulator of CD8+ T-Lymphocyte Activation and Differentiation In Vivo
Source: PLoS One. 2015 Apr 16;10(4):e0124246. doi: 10.1371/journal.pone.0124246 (PMC4399836; doi:10.1371/journal.pone.0124246)
Supplement: S5 Table — The relative levels of expression are highlighted by colour grading according to normalized expression values. The significance of the differential expression values is indicated by the p value of T-test. (PDF) [file pone.0124246.s006.pdf]

S5 Table: Expression levels of iron related genes in $CD8^{+}$ T lymphocytes from *Hfe* knockout and wild type C57BL/6 mice. The relative levels of expression are highlighted by colour grading according to normalized expression values. The significance of the differential expression values is indicated by the *p* value of T-test.

		Normal Iron Diet		
**Gene**	**Protein**	***Hfe*** $^{-/-}$	**C57BL/6**	***p* value**
**Iron storage**				
Ftl1	Ferritin L Chain			ns
FtH	Ferritin Heavy chain			ns
Hmox1	Heme oxigenase			ns
**Iron transport**				
Slc25a37	Mitoferrin			ns
Slc11a2	Dimetal transporter1			ns
Lcn2	Lipocalin2			0.0016
Sfxn2	Sideroflexin2			ns
Slc40a1	Ferroportin			ns
Abcg2	Bcrp			ns
**Receptors**				
Tfrc	Transferrin receptor			ns
Lrp1	LRP/CD91			ns
**Regulators**				
Smad4	Smad4			ns
Smad7	Smad7			ns
Usf2	Usf2			ns
Hamp2	Hepcidin 2			ns
Fxn	Frataxin			ns
Ireb2	IRP2			ns
BMP6	BMP6			ns
BMP9	BMP9			ns
Hamp1	Hepcidin 1			ns
Hfe2	HJV			ns
Hfe	HFE			ns
**Oxidoreductases**				
Cybrd1	Dcytb			ns

- No expression (< 5normalized gene expression value)
- low expression ([5 a 7[normalized gene expression value)
- medium expression([7 a 9[normalized gene expression value)
- high expression(>9 normalized gene expression value)
