# Supplementary material for: Lymphocyte Gene Expression Signatures from Patients and Mouse Models of Hereditary Hemochromatosis Reveal a Function of HFE as a Negative Regulator of CD8+ T-Lymphocyte Activation and Differentiation In Vivo
Source: PLoS One. 2015 Apr 16;10(4):e0124246. doi: 10.1371/journal.pone.0124246 (PMC4399836; doi:10.1371/journal.pone.0124246)
Supplement: S6 Table — (PDF) [file pone.0124246.s007.pdf]

S6 Table: **Sequences of the oligonucleotide primers used for the first and second PCR experiments in CD8 T lymphocytes from HH patients**. Primers on the top: forward primers; Primers on the bottom: reverse primers

Gene	Accession number	1st PCR primers	2nd PCR primers
LEF1	AF288571	5'-ATCCCGAAGAGGAAGGCGATT-3'<br>5'-GCACCACGGGCACTTTATTTG-3'	5'-CCGATGACGGAAAGCATCCAG-3'<br>5'-GCACCACGGGCACTTTATTTG-3'
ACTN1	DQ496098	5'-TGAAGATGACCCTGGGCATGA-3'<br>5'-CAACGATGTCTTCGGCATCCA-3'	5'-GGAAGGATGGCCTCGGCTT-3'<br>5'-CAACGATGTCTTCGGCATCCA-3'
CCR7	BC035343	5'-ACTTCCTCCCCAGACAGGGGT-3'<br>5'-GCCCACGAAACAAATGATGGA-3'	5'-TGGTGGTGGCTCTCCTTGTCA-3'<br>5'-GCCCACGAAACAAATGATGGA-3'
NR4A2	BC009288	5'-CCCGGTGAGTCTGATCAGTGC-3'<br>5'-CAATCCATTCCCCAAAGCCAC-3'	5'-GAGAAGATCCCTGGCTTCGCA-3'<br>5'-CAATCCATTCCCCAAAGCCAC-3'
NAA50	BC012731	5'-AGCTGGGAGATGTGACACCACA-3'<br>5'-GCCGACTCATTGCTGATCTGG-3'	5'-GGCACCTTACCGAAGGCTAGGA-3'<br>5'-GCCGACTCATTGCTGATCTGG-3'
P2RY8	NM_178129	5'-CCTTTGCAAGGTTGCTGGACA-3'<br>5'-AGAGAAGAGGTTGCCCGGGAT-3'	5'-TTCTGCCGCTGCTTCTGCA-3'<br>5'-AGAGAAGAGGTTGCCCGGGAT-3'
FOSL2	NM_005253	5'-GCTCAGGCAGTGCATTCATCC-3'<br>5'-TGCAGCCAGCTTGTTCCTCTC-3'	5'-GCGTGATCAAGACCATTGGCA-3'<br>5'-TGCAGCCAGCTTGTTCCTCTC-3'
GAPDH	M33197	5'-GGTCGGAGTCAACGGATTTGG-3'<br>5'-ATGGTGGTGAAGACGCCAGTG-3'	5'-CAAATTCCATGGCACCGTCAA-3'<br>5'-ATGGTGGTGAAGACGCCAGTG-3'
