# Supplementary material for: Lymphocyte Gene Expression Signatures from Patients and Mouse Models of Hereditary Hemochromatosis Reveal a Function of HFE as a Negative Regulator of CD8+ T-Lymphocyte Activation and Differentiation In Vivo
Source: PLoS One. 2015 Apr 16;10(4):e0124246. doi: 10.1371/journal.pone.0124246 (PMC4399836; doi:10.1371/journal.pone.0124246)
Supplement: S7 Table — (PDF) [file pone.0124246.s008.pdf]

S7 Table: **Sequences of the oligonucleotide primers used for S100a8 and S100a9 expression studies in sorted CD8 from HH patients and controls**

**Gene**	**Primer forward**	**Primer reverse**
18S rRNA human	5`-CGCCGCTAGAGGTGAAATTC-3`	5`-TTGGCAAATGCTTTCGCTC-3`
S100a8 human	5`-GTCTCTTGTCAGCTGTCTTTCA-3`	5`-CCTGTAGACGGCATGGAAAT-3`
S100a9 human	5`-GGAATTCAAAGAGCTGGTGC-3`	5`-TCAGCATGATGAACTCCTCG-3`
